# GEF-H1 Signaling upon Microtubule Destabilization Is Required for Dendritic Cell Activation and Specific Anti-tumor Responses

**DOI:** 10.1016/j.celrep.2019.08.057

**Published:** 2019-09-24

**Authors:** Abhishek S. Kashyap, Laura Fernandez-Rodriguez, Yun Zhao, Gianni Monaco, Marcel P. Trefny, Naohiro Yoshida, Kea Martin, Ashwani Sharma, Natacha Olieric, Pankaj Shah, Michal Stanczak, Nicole Kirchhammer, Sung-Moo Park, Sebastien Wieckowski, Heinz Laubli, Rachid Zagani, Benjamin Kasenda, Michel O. Steinmetz, Hans-Christian Reinecker, Alfred Zippelius

**Affiliations:** 1 Department of Biomedicine, University Hospital Basel and University of Basel, 4031 Basel, Switzerland; 2 Gastrointestinal Unit and Center for the Study of Inflammatory Bowel Disease, Massachusetts General Hospital, Harvard Medical School, Boston, MA 02114, USA; 3 Laboratory of Biomolecular Research, Division of Biology and Chemistry, Paul Scherrer Institut, 5232 Villigen, Switzerland; 4 Medical Oncology, University Hospital Basel, 4031 Basel, Switzerland; 5 University of Basel, Biozentrum, 4056 Basel, Switzerland; 6 Present address: Novartis Institute of Biomedical Research, 4002 Basel, Switzerland; 7 Present address: Vaximm AG, 4057 Basel, Switzerland; 8 These authors contributed equally; 9 Lead Contact

## Abstract

Dendritic cell (DC) activation is a critical step for anti-tumor T cell responses. Certain chemotherapeutics can influence DC function. Here we demonstrate that chemotherapy capable of microtubule destabilization has direct effects on DC function; namely, it induces potent DC maturation and elicits anti-tumor immunity. Guanine nucleotide exchange factor-H1 (GEF-H1) is specifically released upon microtubule destabilization and is required for DC activation. In response to chemotherapy, GEF-H1 drives a distinct cell signaling program in DCs dominated by the c-Jun N-terminal kinase (JNK) pathway and AP-1/ATF transcriptional response for control of innate and adaptive immune responses. Microtubule destabilization, and subsequent GEF-H1 signaling, enhances cross-presentation of tumor antigens to CD8 T cells. In absence of GEF-H1, anti-tumor immunity is hampered. In cancer patients, high expression of the GEF-H1 immune gene signature is associated with prolonged survival. Our study identifies an alternate intracellular axis in DCs induced upon microtubule destabilization in which GEF-H1 promotes protective anti-tumor immunity.

## In Brief

Certain chemotherapeutics elicit potent anti-tumor immunity. Kashyap et al. demonstrate that microtubule-destabilizing chemotherapeutics induce maturation of dendritic cells through activation of microtubule-associated protein GEF-H1. This leads to effective priming of CD8 T cells against tumor antigens. GEF-H1 is critical for anti-tumor immunity of microtubule-targeting chemotherapy.

## Graphical Abstract

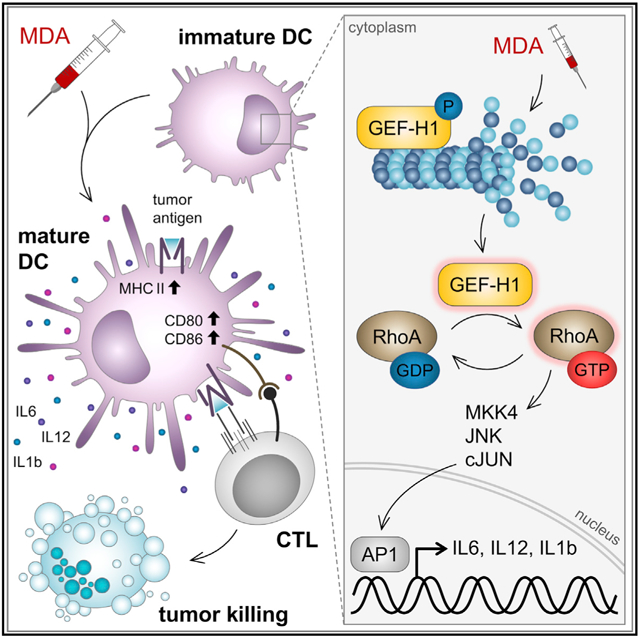

## INTRODUCTION

Because of their efficient antigen processing and presentation machinery, antigen-presenting cells, such as dendritic cells (DCs), play a central role in the initiation and regulation of specific anti-tumor immunity (Melief, 2008). DC maturation is necessary for antigen processing and to provide costimulatory signals to T cells (Mildner and Jung, 2014). Although DC maturation may occur in tumors, it is often insufficient to induce potent immunity and hindered by suppressive mechanisms within tumors (Corrales et al., 2017). Furthermore, in contrast to mature or activated DCs, immature DCs are tolerogenic, are immunosuppressive, and lead to deficient anti-tumor immunity (Gardner and Ruffell, 2016). Bypassing suppressive pathways or directly activating DCs can unleash adaptive immunity through cross-presentation of tumor antigen to generate tumor-specific T cell responses (Wei et al., 2018). Hence, the therapeutic targeting of DC maturation or activation processes is a promising strategy to enhance anti-tumor immunity.

DC maturation is conventionally known to be a consequence of the engagement of pattern recognition receptors (PRRs, including Toll-like receptors [TLRs] and nucleotide-binding domain, leucine rich containing [NLRs]) and/or the CD40-CD40L axis (Kawai and Akira, 2011; Gardner and Ruffell, 2016). The perturbation of microtubules has emerged as an exciting and promising medical concept that potently triggers DC maturation (Müller et al., 2015). As a therapeutic consequence, the targeted delivery of microtubule-destabilizing agents (MDAs) can induce potent anti-cancer adaptive immunity, which can be boosted by immune checkpoint inhibitors. Specifically, antibody drug conjugates (ADCs) incorporating MDAs, such as the maytansine DM1 (trastuzumab emtansine) or the auristatin monomethyl auristatin E (MMAE) (brentuximab vedotin), activate DCs (Müller et al., 2014a, 2015) and are of high clinical relevance (Verma et al., 2012; von Minckwitz et al., 2019; Younes et al., 2010; Connors et al., 2018). This DC activation enhances the capture of tumor antigens and the production of proinflammatory cytokines, which improves the intra-tumoral infiltration of tumor antigen-specific effector T cell populations and therapeutic synergy with immune checkpoint inhibitors (Müller et al., 2015). MDAs administered as free drugs, such as vinblastine (Tanaka et al., 2009), colchicine (Mizumoto et al., 2007), ansamitocin-P3 (Martin et al., 2014), and dolastatin-10 (Müller et al., 2014a), have a similar capacity to induce DC maturation and T cell-dependent tumor control. However, the distinct immune activation pathways in DCs operational downstream of microtubule destabilization remain elusive.

Guanine nucleotide exchange factor-H1 (GEF-H1), encoded by the *Arhgef2* gene, is a member of the Dbl family of guanine nucleotide exchange factors (GEFs) that is sequestered on microtubules (Meiri et al., 2012), and is linked to the activation of Rho guanosine triphosphatases (GTPases) (Krendel et al., 2002). GEF-H1 is implicated in numerous cellular processes, such as cell motility and polarization (Fine et al., 2016), cell-cycle regulation, epithelial barrier permeability, and cancer (Birkenfeld et al., 2008). GEF-H1 contributes to immune signaling in macrophages during anti-viral host defense responses (Chiang et al., 2014) and intracellular pathogen recognition (Zhao et al., 2012, 2019; Fukazawa et al., 2008). How GEF-H1 is released and controls cellular functions in response to changing microtubule dynamics, especially in antigen-presenting cells, remains unclear as yet.

Here, we investigated the consequence of perturbing microtubule dynamics in DCs and focus on the distinct downstream molecular and cellular mechanisms that control DC maturation and antigen presentation to T cells. Collectively, we identify GEF-H1 as a key alternate axis in DC maturation, which is induced after microtubule destabilization. We found that through the microtubule release of GEF-H1, MDAs can induce immune responses that normally require host defense activation by microbial PRRs. Activation of GEF-H1 signaling by MDAs induced cross-presentation of antigens to drive specific CD8 T cell responses during anti-cancer chemotherapy.

## RESULTS

### Microtubule Destabilization Leads to Phenotypic and Functional Maturation of DCs

**Figure 1.**
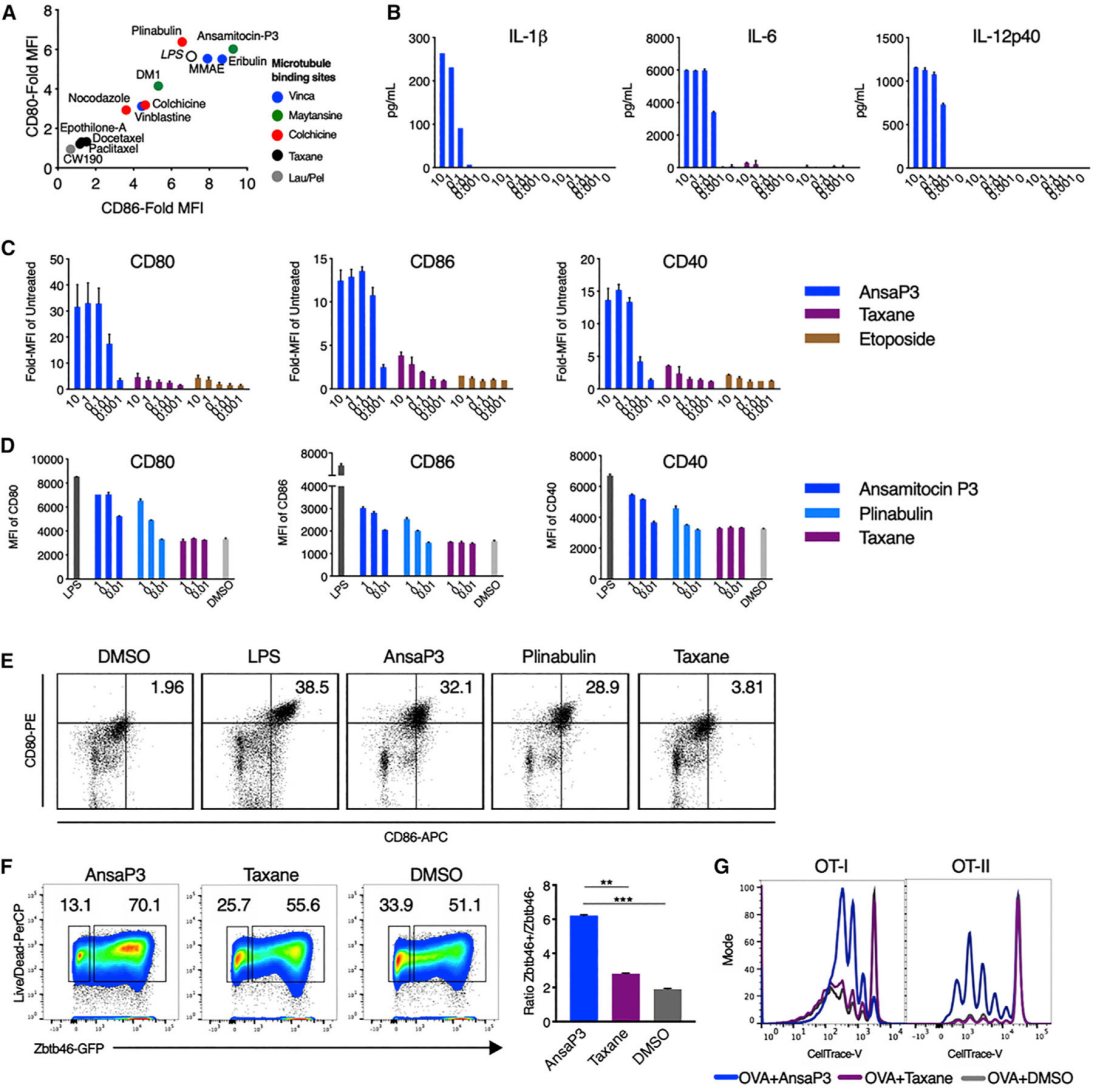
Microtubule Destabilization, but Not Stabilization, Induces DC Maturation (A) SP37A3 cells were treated with various drugs at 100 nM or LPS at 500 ng/mL. CD80 and CD86 expression was assessed after 20 h using flow cytometry and expressed as fold-mean fluorescence intensity (MFI) of 0.1% DMSO. n = 3 biological replicates. (B) Quantification of cytokines (in picograms per milliliter) using ELISA from supernatant of SP37A3 cells treated for 20 h at indicated concentrations (in micromolars). n = 2 biological replicates. (C) Surface expression of CD80, CD86, and CD40 on cells from (B) was assessed using flow cytometry. (D) Splenic DCs from C57BL/6N mice were treated with LPS (200 ng/mL), ansamitocin-P3, plinabulin, and taxane at indicated doses (in nanomolars), or 0.1% DMSO. The MFI of CD80, CD86, and CD40 was assessed after 20 h by flow cytometry. n = 2 biological replicates. (E) Dot plots and percentage of CD80 and CD86 double-positive cells from live CD11c^+^MHC-II^+^ DCs from (D) are depicted. Representative plots from four biological replicates are indicated. (F) BMDCs from Zbtb46-GFP mice were cultured with ansamitocin-P3, taxane, or 0.1% DMSO for 24 h, and Zbtb46 expression (GFP) was assessed by flow cytometry (gating: CD11c^+^MHCII^+^GFP^+^). The bar graph represents the ratio of Zbtb46^hi^ versus Zbtb46^low^ cells. **p < 0.01, ***p < 0.001; n = 3 mice. (G) SP37A3 cells pretreated with 100 nM ansamitocin-P3, taxane, or 0.1% DMSO were pulsed with OVA protein and cocultured with OT-I (1:20 DC:T cell) or OT-II (1:15 DC:T cell) T cells labeled with CellTrace violet dye. Dye dilution in OT-I/OT-II cells was assessed using flow cytometry after 72 h. Representative overlapping histograms are presented. Experiment was repeated three times with similar results. Error bars represent SD. See also Figure S1.

MDAs administered as free drugs or delivered as ADCs boost anti-tumor immune responses by inducing the full spectrum of DC maturation and the release of proinflammatory cytokines (Martin et al., 2014; Müller et al., 2014b). To confirm a class effect of microtubule-targeting agents, we tested various MDAs and microtubule-stabilizing agents (MSAs) for their capacity to induce DC maturation based on the upregulation of cell surface CD80 and CD86. The MDAs ansamitocin-P3, MMAE, plinabulin, and eribulin all potently induce activation of the immature DC cell line SP37A3. In contrast, the MSAs epothilone-A and peloruside derivative CW190, as well as taxanes, namely, docetaxel and paclitaxel, had no DC-stimulatory effects (Figure 1A; Figure S1A). The targeting of different tubulin-binding sites by MDAs did not correlate with the potency of DC activation (Figure 1A).

Treatment of SP37A3 cells with ansamitocin-P3 induced significant production of proinflammatory cytokines interleukin(IL)-1β, IL-6, and IL-12 at doses greater than 100 nM (Figure 1B). In addition, exposure to ansamitocin-P3 induced the expression of the costimulatory molecules CD80, CD86, and CD40 (Figure 1C; Figure S1B). The dosing used for the MDAs favorably compares with the dosing used in clinics (patient dosing data available for plinabulin and vincristine; Mita et al., 2010; Yang et al., 2018). DC viability was not reduced compared with vehicle at all concentrations of ansamitocin-P3 tested (Figure S1C). Taxane and etoposide (a topoisomerase inhibitor that does not target microtubules) did not induce DC maturation (Figures 1A–1C), indicating specificity to MDAs. Moreover, this indicates that microtubule destabilization was sufficient for DC maturation even in the absence of PRR ligands such as lipopolysaccharide (LPS). Similar induction of DC maturation was observed in freshly isolated splenic DCs specifically upon exposure to MDAs ansamitocin-P3 and plinabulin in a dose-dependent manner and was comparable to LPS-induced DC maturation (Figures 1D and 1E; Figure S1D). Furthermore, ansamitocin-P3 treatment of bone marrow-derived DCs (BMDCs) from Zbtb46-GFP reporter mice led to the differentiation of classical DCs (cDCs), as measured by the induction of the transcription factor Zbtb46 (Satpathy et al., 2012) (Figure 1F). Accordingly, taxane had no effect on promoting cDC differentiation (Figure 1F).

To assess the activation of antigen-specific T cell responses, SP37A3 cells were pretreated with ansamitocin-P3 or taxane, loaded with ovalbumin (OVA) and cocultured with labeled CD8 and CD4 T cells isolated from OT-I and OT-II T cell receptor (TCR) transgenic mice, respectively. Treatment of DCs with ansamitocin-P3, but not taxane, led to robust CD8 and CD4 T cell proliferation (Figure 1G). This suggested that microtubule destabilization alone promotes DC maturation, leading to both major histocompatibility complex (MHC) class I and MHC class II antigen presentation.

### Microtubule Destabilization by MDAs Releases and Activates GEF-H1

**Figure 2.**
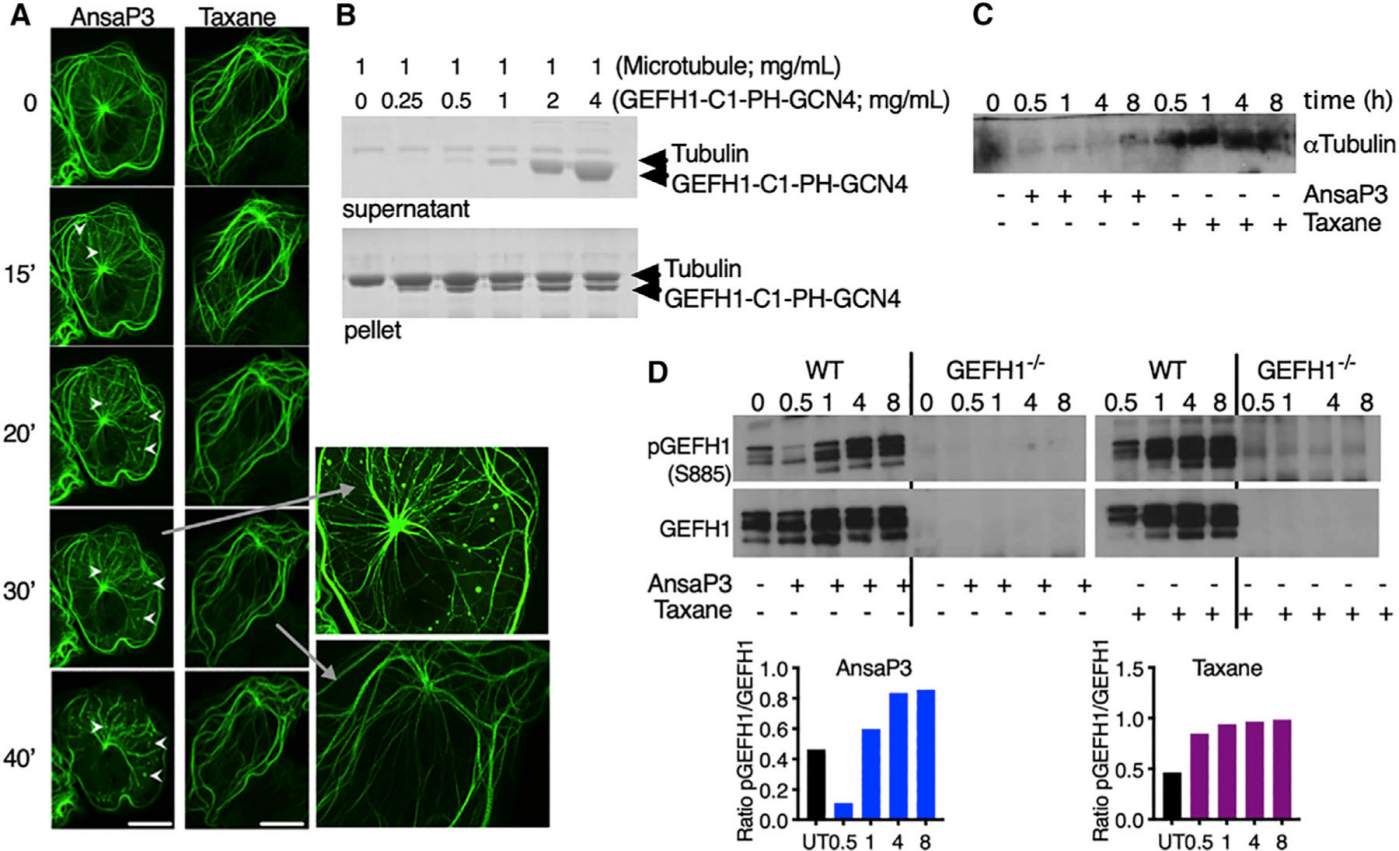
GEF-H1 Release and Activation upon Microtubule Destabilization (A) COS-7 fibroblasts were transfected with GEF-H1-GFP plasmid and imaged upon treatment with 1 μM ansamitocin-P3 or taxane using confocal live cell microscopy. Time is depicted in minutes. Arrowheads indicate GEF-H1 delocalization. Scale bar, 40 μm. (B) Coomassie-stained SDS-PAGE showing the cosedimentation of microtubules (1 mg/mL) with increasing concentration of GEF-H1-C1-PH-GCN4 (upper blot, supernatant fractions; lower blot, pellet fractions). (C) GEF-H1 was immunoprecipitated from WT BMDCs treated with ansamitocin-P3 or taxane (100 nM) for indicated time points (in hours) and was probed for α-tubulin. (D) Lysates obtained from (C) were probed for phosphorylated and total GEF-H1. GEF-H1 activation was quantified using densitometry and depicted as the ratio of phosphorylated GEF-H1 (pGEF-H1) to total GEF-H1. The experiment was repeated twice with comparable results. See also Figure S2 and Video S1.

Microtubule-associated GEF-H1 can initiate intracellular signaling, leading to the release of proinflammatory cytokines in macrophages (Chiang et al., 2014). We therefore investigated whether GEF-H1 was responsible for DC maturation upon microtubule destabilization. Using COS-7 fibroblasts overexpressing GEF-H1-GFP, we demonstrated the release of GEF-H1 from the microtubule network as early as 15 min upon treatment with ansamitocin-P3 (Figure 2A, arrowheads; Video S1). The release of GEF-H1 did not occur upon microtubule stabilization by taxane (Figure 2A). GEF-H1 is reported to bind to microtubules through interaction with the dynein motor complex (Meiri et al., 2012).

It has been proposed that the zinc-finger motif-containing C1 domain, the pleckstrin homology (PH) domain, and the coiled-coil domain of GEF-H1 are involved in microtubule binding (Krendel et al., 2002; Glaven et al., 1999). To test the possibility that GEF-H1 (Figure S2A) binds directly to microtubules, we sought to perform a biochemical experiment with purified proteins. We thus cloned a construct in which we fused the C1, PH, and the coiled-coil domain of GCN4 (denoted GEF-H1-C1-PH-GCN4) (see STAR Methods). Using a standard *in vitro* microtubule pelleting assay, we demonstrate that GEF-H1-C1-PH-GCN4 binds in a specific manner to microtubules (Figure 2B; Figure S2B). This finding suggests that GEF-H1 can interact directly with microtubules and is released from this binding upon treatment with MDAs.

The MDA-specific release of GEF-H1 from microtubules was subsequently confirmed in BMDCs treated with ansamitocin-P3 using coimmunoprecipitation. A decreased amount of a-tubulin observed in western blotting was correlated with reduced binding of GEF-H1 to microtubules (Figure 2C). Furthermore, ansamitocin-P3, but not taxane, treatment of BMDCs rapidly dephosphorylated GEF-H1 within 30 min (Figure 2D), a critical step associated with the activation and release of GEFH1 from microtubules (Meiri et al., 2012; Chiang et al., 2014). GEF-H1 re-phosphorylated within 60 min of treatment with ansamitocin-P3, suggesting the involvement of certain kinases that need to be further investigated. Lack of phosphorylated and total GEF-H1 was noted in BMDCs of GEF-H1-deficient (GEF-H1^−/−^) mice (Figure 2D). GEF-H1 activation is known to be accompanied by the activation of Ras homolog gene family, member A (RhoA)-guanosine diphosphate (GDP) (Matsuzawa et al., 2004). The transient activation of GEF-H1 was observed to lead to the accumulation of RhoA-guanosine triphosphate (GTP) within 30 min of ansamitocin-P3 treatment (Figures S2C and S2D). The inhibition of RhoA using CCG-1423 prevented ansamitocin-P3-induced DC activation in a dose-dependent manner (Figure S2E).

Ansamitocin-P3 treatment of BMDCs derived from TLR4^−/−^, TRIF^−/−^, and NALP3^−/−^ mice demonstrated that DC maturation in response to microtubule disruption occurred independent of TLR4, TRIF−/−, or NLRP3 inflammasome activation (Figures S2F–S2H). Altogether, destabilization of microtubules was sufficient to induce potent DC maturation, wherein GEF-H1 release induced a potent downstream signaling pathways to promote DC subspecification and maturation.

### GEF-H1-Dependent Transcriptional Programs Signal Microtubule Destabilization for the Activation of DCs

**Figure 3.**
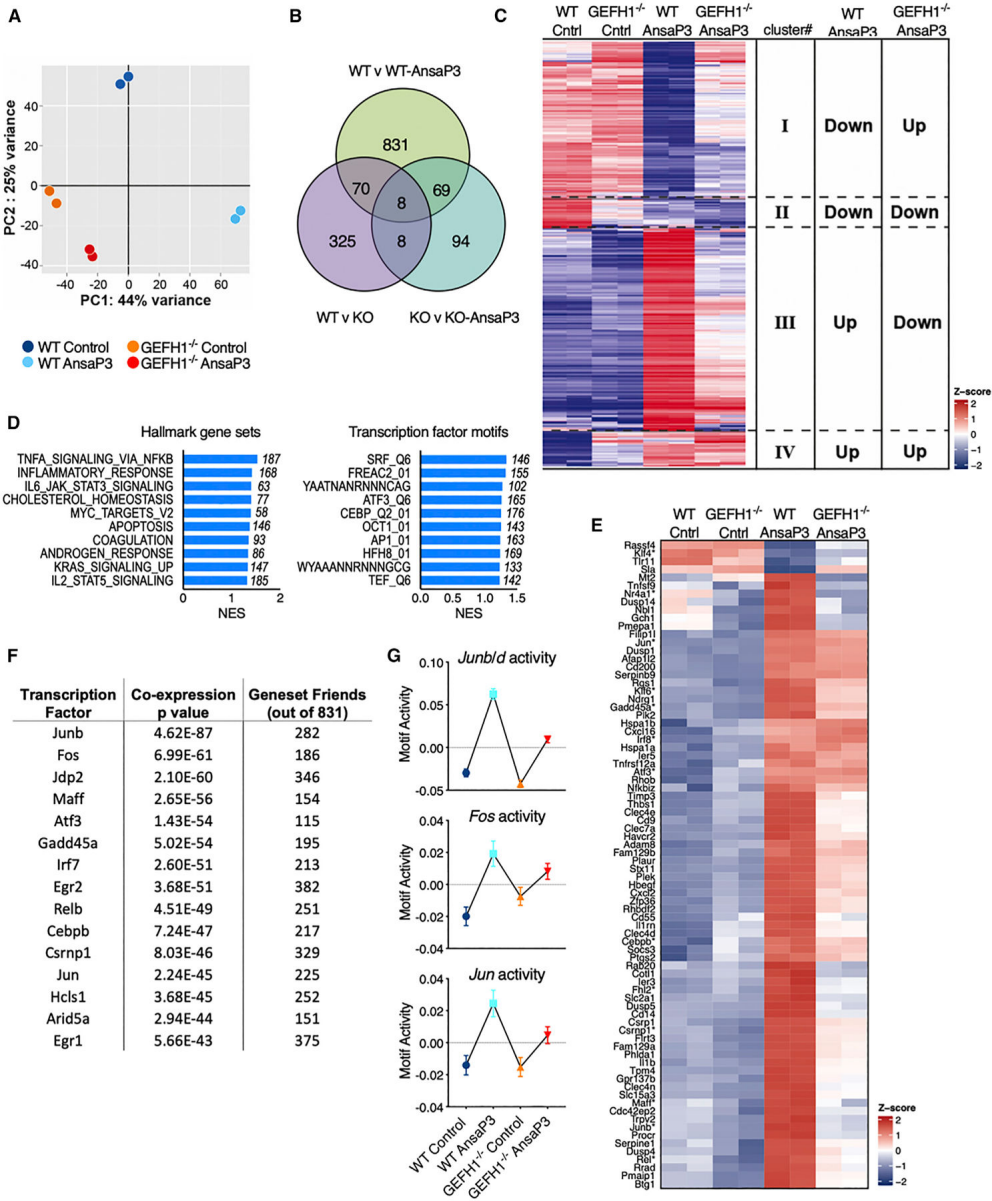
Transcriptional Profiling of WT and GEF-H1-Deficient BMDCs Subjected to Microtubule Destabilization (A) Principal component analyses of expression values color coded by treatment groups. (B) Venn diagram of differentially expressed genes in indicated pairwise comparisons (false discovery rate [FDR] < 0.05 and log fold change [logFC] > 1). Knockout (KO) denotes GEF-H1^−/−^ BMDCs. (C) Heatmap of genes differentially expressed (p < 0.01, FDR < 0.05, and logFC > 1) in WT BMDCs treated with and without ansamitocin-P3 represented across all indicated samples (duplicates per sample). Hierarchical clustering separated genes into 4 clusters. These were either GEF-H1 dependent (clusters I and III) or GEF-H1 independent (clusters II and IV). (D) Top gene sets enriched in the GEF-H1-dependent ansamitocin-P3 treatment response performed using the Broad Institute GSEA method for the Hallmark and C3 transcription factor motif gene set collections. Shown are the top 10 gene sets containing at least 50 overlapping genes ordered by their normalized enrichment scores (NESs). The number of overlapping genes within each gene set is indicated. (E) Top 80 genes and their scaled, centered log fragments per kilobase million (logFPKM) values selected from the gene signature comprising cluster I and III in (C) retrieved from the coexpression enrichment analysis using GeneFriends. Asterisks indicate transcription factors. F) Top 15 transcription factors that are coexpressed with the gene signature of (E) were mapped using GeneFriends. In all cases, heatmaps indicate scaled, centered logFPKM values across all samples. (G) ISMARA analyses of transcription factor motif activity across the four samples. JunB, JunD, Jun, and Fos were the top regulated transcription factors. Error bars represent SD. See also Figure S3 and Tables S1, S2, S3, and S4.

To gain insights into the GEF-H1-dependent molecular mechanisms activated upon destabilization of microtubules, we performed high-resolution RNA sequencing (RNA-seq). We used duplicate samples of RNA isolated from BMDCs of GEF-H1^−/−^ and wild-type (WT) mice pretreated for 5 h with ansamitocin-P3. Microtubule destabilization induced a significant GEF-H1-dependent inflammatory response with the expression of genes such as *Il1a*, *Il1b*, *Il6*, *cd80*, *cd14*, and chemokines associated with nuclear factor κB (NF-κB)/AP-1 activation (Table S1). This gene signature was synonymous with innate immune activation in response to microbial stimuli. Principal component analysis (PCA) of normalized expression revealed that control and ansamitocin-P3-treated WT DCs segregate into distinct quartiles, whereas the control and treated DCs lacking GEF-H1 remained in the same quartile (Figure 3A). The lack of transcriptional changes in GEF-H1 lacking DCs was also revealed in pairwise comparisons, in which GEF-H1^−/−^ DCs lack most ansamitocin-P3-induced transcriptional changes (Figure 3C). Furthermore, hierarchical clustering (Seqmonk; Babraham Bioinformatics) of ansamitocin-P3-regulated genes revealed that a significant proportion of the ansamitocin-P3-induced transcriptional response required GEF-H1 (Figure 3C). Of the 984 regulated genes with more than 2-fold upon microtubule destabilization in WT DCs (also seen in Figure 3B), GEF-H1 was required for inhibition of 362 or induction of 469 transcripts (Figure 3C, clusters I and III; Table S2). This suggested that changes in gene expression occurring downstream of microtubule destabilization critically depended on the presence of GEF-H1. Nevertheless, we detected minor proportion of GEF-H1-independent changes to the destabilization of microtubules within two additional clusters of 68 and 81 transcripts (Figure 3C, clusters II and IV; Table S2) that remained either decreased or elevated in both WT or GEF-H1^−/−^ treated DCs (Figure 3C).

For gene set enrichment analyses (GSEAs) of GEF-H1-dependent transcriptional activation, genes were ranked on their dependence on GEF-H1 and their extent of regulation upon microtubule destabilization. GSEAs revealed that GEF-H1 controlled a microtubule destabilization-induced innate immune transcriptional signature normally associated with proinflammatory host defenses. The top three significant Hallmark biogroups included tumor necrosis factor alpha (TNF-α) signaling (overlap of 187 genes; normalized enrichment score [NES] = 1.53), inflammatory response (overlap of 168 genes; NES = 1.42), and IL-6-JAK-STAT3 signaling (overlap of 77 genes; NES = 1.40) (Figure 3D; Figure S3A). These contain major innate immune regulators such as *Il1a*, *Il1b*, *Il6*, *cd80*, *tnfsf4*, *tnfsf15*, *nfkb1*, *jun*, and the GEF-H1 interactor *ripk2* (Figure S3A). The GEF-H1-dependent genes significantly enriched for the transcription factor motif biogroup of ATF3 (overlap of 165 genes; NES = 1.26), CEBPB (overlap of 176 genes; NES = 1.25), AP-1 (overlap of 163 genes; NES = 1.23), and serum response factor (SRF)-binding site gene sets (Figure 3D; Figure S3B). Both AP-1 (dimer of c-Jun/c-Fos) and CEBPB (interacts with c-Jun, c-Fos, and NF-κB) belong to the activating transcription factor (ATF) family of transcription factors and are predominantly involved in the regulation of proinflammatory responses (Huber et al., 2012; Thompson et al., 2009).

To retrieve the most pivotal and central genes within the GEF-H1-dependent gene signature (Figure 3C, clusters I and III) (831 genes), we performed coexpression enrichment analysis (van Dam et al., 2012). The genes were ranked according to their overall coexpression within the signature, and the top 80 genes were selected, expression of which across treatments was represented as a heatmap in Figure 3E (and Table S3). The selected genes were assumed to be the central and most fundamental genes involved in the GEF-H1 signaling program in response to MDAs. Using coexpression analyses, we also mapped the top 15 transcription factors coexpressed with these 831 genes (Figure 3F). The top 3 belonged to the AP-1/ATF family, which also confirmed the results obtained with GSEAs (Figure 3D) in this independent and unbiased analysis. In addition, we performed an integrated system for motif activity response analysis (ISMARA) to determine the activity of transcription factor motifs in a genome-wide analysis (Balwierz et al., 2014). This analyses revealed JunB/Junc/Fos transcription factors (AP-1 transcription factor complex) are the dominant GEF-H1-dependent signaling output of ansamitocin-P3 (Figure 3G; Table S4). Altogether, the transcriptome analyses of BMDCs treated with MDA revealed that GEF-H1 controlled most proinflammatory gene expression signatures that signaled microtubule destabilization in DCs.

### Microtubule Destabilization and Release of GEF-H1 Leads to c-Jun and Interferon Response Factor (IRF) Activation

**Figure 4.**
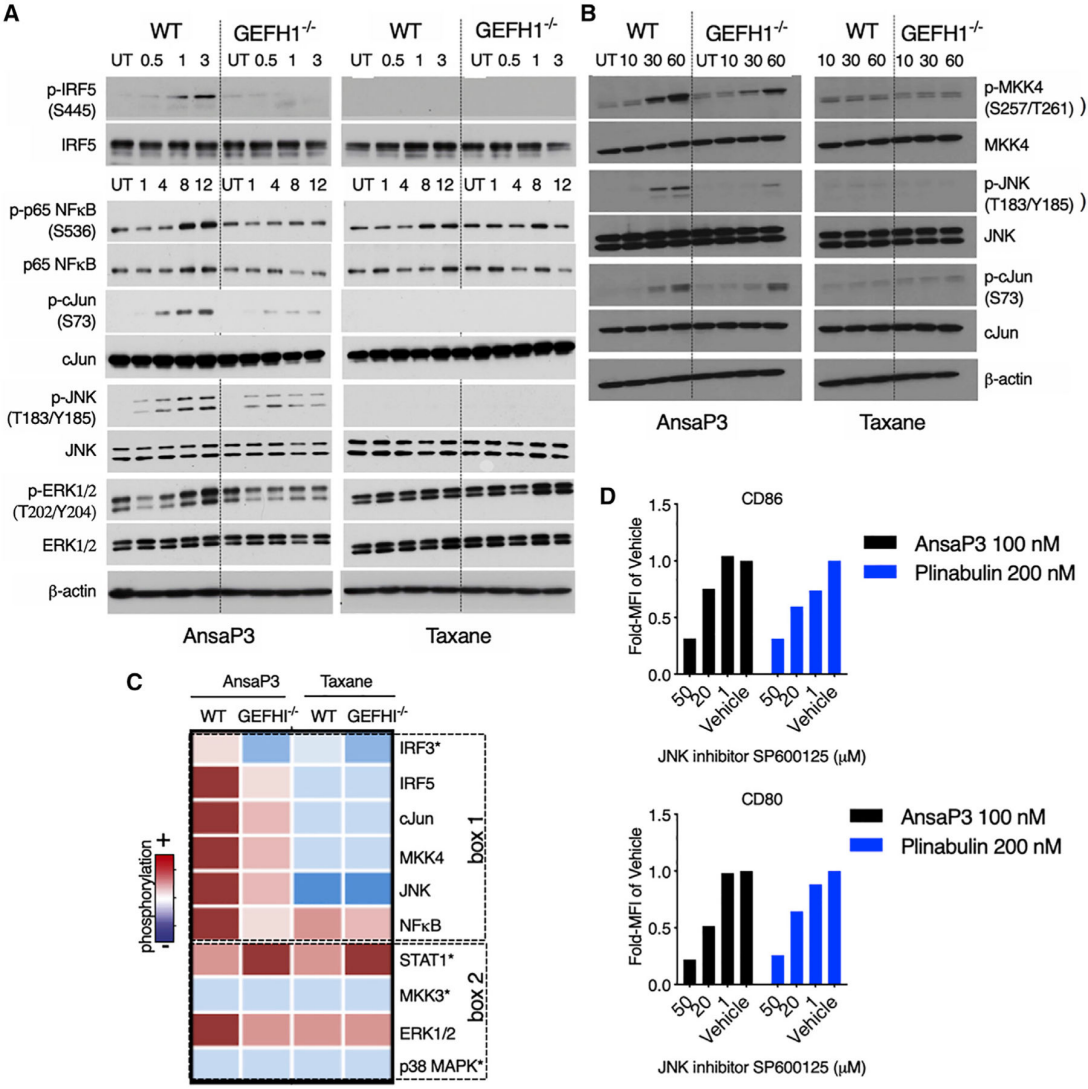
Differential Activation of Cell Signaling Intermediates upon Microtubule Destabilization and Stabilization (A and B) Lysates from WT or GEF-H1^−/−^ BMDCs treated for specified time points with ansamitocin-P3 or taxane (both 100 nM) were probed for the indicated phosphorylated proteins. Time points are indicated in hours in (A) and in minutes in (B). Blots were stripped and re-probed for the respective total proteins. (C) Qualitative intensity map of phosphorylation profile (from A and B) of the various signaling intermediates is represented across the outlined BMDC samples. Box 1 represents signaling intermediates activated uniquely in response to ansamitocin-P3 in a GEF-H1-dependent manner. Non-specifically activated or nonactivated proteins are represented in box 2. Blots with an asterisk are in Figure S4. (D) DCs were preincubated with the indicated concentrations of the JNK inhibitor SP600125 or vehicle (0.5% DMSO) for 2 h, after which they were exposed to MDAs ansamitocin-P3 (100 nM) or plinabulin (200 nM) for 20 h. Data are represented as fold change in MFI of CD80 and CD86 compared with vehicle-treated cells. n = 3 technical replicates. The experiment was performed twice with similar results. See also Figure S4.

To identify the precise signaling events that mediate GEF-H1-dependent immune activation, we assessed the activation status of key transcription factors (IRF3, IRF5, STAT1, p65 NF-κB, and c-Jun) and cell signaling intermediates (ERK1/2, c-Jun N-terminal kinase [JNK], and p38 mitogen-activated protein kinase [MAPK]) in WT and GEF-H1^−/−^ BMDCs. We found GEF-H1 is required for the activation of the transcription factors c-Jun, p65 NF-κB, IRF3, and IRF5 and the signaling intermediates JNK and ERK1/2 upon ansamitocin-P3-induced microtubule destabilization (Figures 4A and 4C; Figure S4A). The activation of IRF5, c-Jun, and JNK by GEF-H1 specifically occurred as a consequence of microtubule destabilization. In contrast, stabilization of microtubules by taxane resulted in GEF-H1-independent activation of STAT1, NF-κB, and ERK1/2 (Figures 4A and 4C; Figure S4A). The cellular response to ansamitocin-P3 was further characterized by the GEF-H1-dependent activation of MKK4, an upstream kinase for JNK activation (Figures 4B and 4C). MKK3, which is not involved in the activation of JNK (Dérijard et al., 1995), remains inactive in response to ansamitocin-P3 (Figure S4A). Microtubule stabilization by taxane did not activate either MKK3 or MKK4. We found the activation of the JNK pathway was critical for DC maturation, because the JNK inhibitor SP600125 blocked CD80 and CD86 expression in response to stimulation with the MDAs ansamitocin-P3 and plinabulin (Figure 4D; Figure S4B). Altogether, we found that microtubule destabilization initiated profound innate immune responses in DCs that normally signal innate immune activation for host defenses.

### GEF-H1 Signaling Is Required for DC Maturation upon Microtubule Destabilization

**Figure 5.**
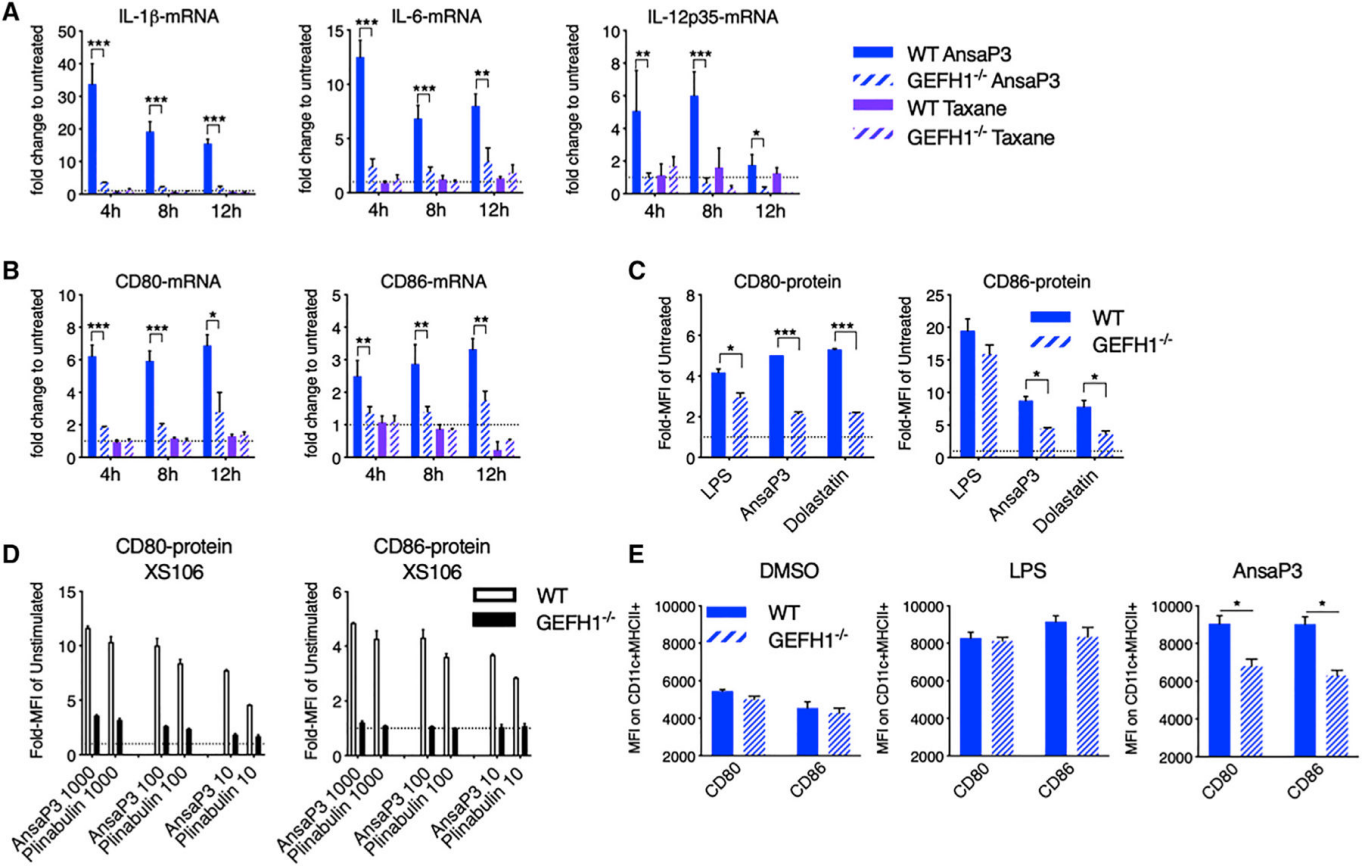
Involvement of GEF-H1 in Microtubule Destabilization-Induced DC Activation (A–C) WT and GEF-H1^−/−^ BMDCs treated with ansamitocin-P3 or taxane (both 100 nM) were assessed for expression of cytokines and DC activation markers using qPCR (A and B) at indicated time points or using flow cytometry (C) 20 h after treatment. (D) CD80 and CD86 expression was assessed by flow cytometry in WT or GEF-H1^−/−^ XS106 cells treated at indicated doses (in nanomolars) for 20 h. (E) Ansamitocin-P3 (4 μg), LPS (8 μg), or vehicle alone (1.5% DMSO) was injected in the earflaps of WT and GEF-H1^−/−^ mice. CD80 and CD86 expression after 20 h on *in situ* intradermal CD11c^+^MHC-II^+^ DCs was analyzed by flow cytometry. In all cases, asterisks indicate statistical comparison between WT and GEF-H1^−/−^. *p < 0.05, **p < 0.01, ***p < 0.001. Data in (A)–(D) are from three biological repeats and in (E) are from two biological repeats (technical repeats R 6). Error bars represent SD. See also Figure S5.

We next determined whether GEF-H1-mediated signals were responsible for directing DC function in response to microtubule destabilization. Compared with WT, GEF-H1^−/−^ BMDCs stimulated with ansamitocin-P3 failed to induce mRNA expression of cytokines *Il1b*, *Il6*, and *Il12a* (Figure 5A) and costimulatory molecules CD80 and CD86 (Figure 5B). Both WT and GEF-H1^−/−^ BMDCs failed to mature in response to the MSA taxane (Figure 5B). DC maturation that occurred in response to an additional MDA, dolastatin-10, also depended on GEF-H1 (Figure 5C; Figure S5A). As an additional control, we generated a XS106 DC cell line lacking GEF-H1 expression by CRISPR/Cas9 targeting. In the absence of GEF-H1, CD80 and CD86 protein expression remained uninduced in response to MDAs ansamitocin-P3 as well as plinabulin (Figure 5D; Figure S5B), even over extended periods of up to 72 h (Figures S5C and S5D).

To assess *in vivo* DC maturation upon microtubule destabilization, we injected ansamitocin-P3, LPS, or vehicle (DMSO) into the earflap of WT and GEF-H1^−/−^ mice. In WT mice, ansamitocin-P3 induces significantly higher expression of CD80 and CD86 in isolated DCs compared with GEF-H1^−/−^ mice (Figure 5E; Figure S5E). However, GEF-H1 absence had minimal impact on LPS-induced DC activation *in vivo* (Figure 5E). Altogether, our results indicated that GEF-H1 is required for the maturation of DCs by MDAs that facilitate microtubule polarization.

### GEF-H1 Signaling Controls CD8 T Cell Activation upon DC Maturation by MDAs

**Figure 6.**
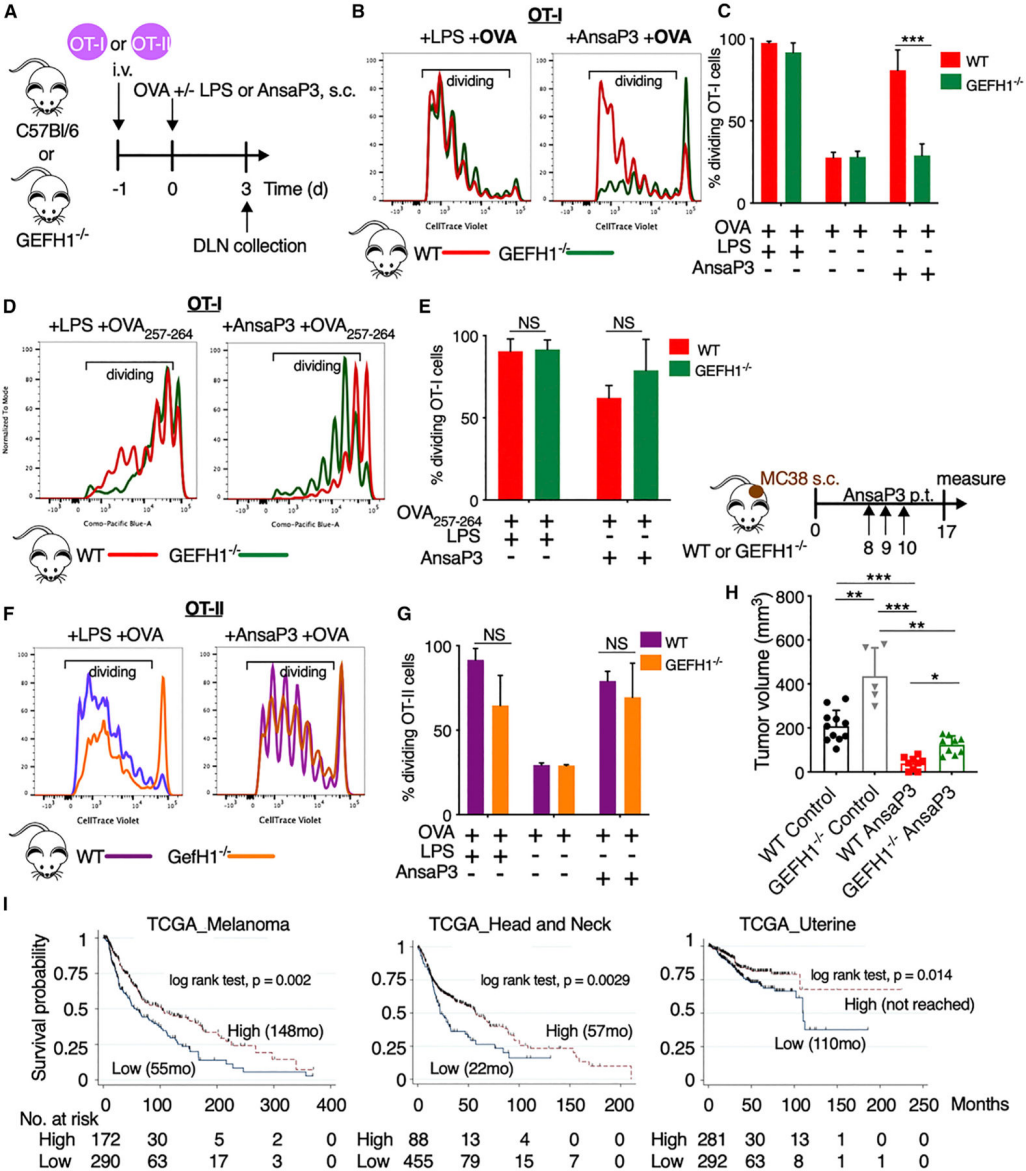
Assessment of GEF-H1 in T Cell Expansion and Anti-tumor Immunity (A) Experimental setup for (B)–(G). CellTrace violet-labeled CD8/CD4 T cells of OT-I/OT-II transgenic mice, respectively, were adoptively transferred into WT or GEF-H1^−/−^ recipient mice. After 24 h, mice were immunized with 25 μg OVA or the OT-I OVA_257–264_ peptide (SIITFEKL) via tail base in the presence of ansamitocin-P3 (4 μg/mouse), LPS (25 μg/mouse), or vehicle (0.5% DMSO). Proliferation of donor-derived OT-I CD8 and OT-II CD4 T cells was assessed by flow cytometry 3 days after immunization. (B, D, and F) Representative histograms indicate overlap of CellTrace violet dye dilution of donor OT-I (B and D) or OT-II (F) T cells isolated from draining lymph nodes (DLNs) of WT and GEF-H1^−/−^ recipient mice. (C, E, and G) Percentage of proliferating (dividing) OT-I (C and E) and OT-II (G) is calculated based on events within the gates as per (B), (D), and (F). ns, not significant (p > 0.05), ***p < 0.001. Data are obtained from three biological repeats (n = 9 mice). (H) Tumor volume (at day 17 after cell injection) of MC38 tumor-bearing WT or GEF-H1^−/−^ mice after peri-tumoral (p.t.) injection (on days 8, 9, and 10) of vehicle (2% DMSO) or ansamitocin-P3 (0.3 mg/kg). Only animals bearing homogeneous tumors across all groups (between 50 and 70 mm^3^) before treatment start were included in the experiment. *p < 0.05, ***p < 0.001. Each data point represents a mouse. (I) Kaplan-Meier survival plot from TCGA analyses in patients stratified by the GEF-H1 immune signature high and low based on the cutoff of 14 log2 FPKM as per Figure S6C (high, median log2 FPKM > 14; low, median log2 FPKM < 14). The number of patients at risk within the stratified groups is depicted at each time point. Error bars represent SD. See also Figure S6 and Table S5.

We next determined the role of GEF-H1 signaling in DCs for the induction of antigen-specific T cell responses. We adoptively transferred labeled CD8 and CD4 T cells, respectively, isolated from OT-I and OT-II transgenic mice, into congenic WT or GEF-H1^−/−^ recipient mice. We measured the proliferation of T cells in the draining lymph node following immunization with ansamitocin-P3 or LPS in the presence of full-length OVA protein (Figure 6A). In WT animals, ansamitocin-P3 was as potent as LPS in significantly enhancing OT-I (Figures 6B and 6C) and OT-II (Figures 6F and 6G) T cell proliferation. Similar effects for WT BMDCs are observed *in vitro* (Figures S6A and S6B). However, we noticed a profound reduction of proliferating, adoptively transferred OT-I T cells in GEF-H1^−/−^ mice after immunization with ansamitocin-P3, although GEF-H1^−/−^ mice were able to sustain LPS-induced OT-I T cell proliferation (Figures 6B and 6C). This selective effect on CD8 T cell proliferation in GEFH1^−/−^ mice, suggesting deficits in antigen cross-presentation, was confirmed *in vitro* using coculture experiments of OT-I CD8 T cells with BMDCs derived from GEF-H1^−/−^ and WT mice (Figures S6A and S6B). To specifically investigate the impact of GEF-H1 on antigen processing versus antigen presentation during cross-priming of CD8 T cells, we immunized ansamitocin-P3- or LPS-treated GEF-H1^−/−^ and WT mice with the OT-I OVA257–264 peptide (Daniels et al., 2006) (Figures 6D and 6E). Upon peptide immunization, OT-I CD8 T cells were equally proliferative in both WT and GEF-H1^−/−^ mice treated with ansamitocin-P3. This suggests that the intracellular antigen processing machinery of antigen cross-priming, not the extracellular antigen presentation, requires intact GEF-H1 signaling. Altogether, these data indicated that GEF-H1 was specifically required for efficient MHC class I-mediated CD8 T cell activation, because OT-II cells still underwent substantial proliferation after immunization with ansamitocin-P3 or LPS in GEF-H1^−/−^ mice (Figures 6F and 6G).

### GEF-H1 Signaling Controls Ectopic Tumor Growth and Promotes Anti-tumor Immunity of MDAs

We next investigated the role of GEF-H1 in tumor rejection. It is known that ansamitocin-P3 treatment of immunocompetent C57BL/6N WT mice bearing MC38 tumors leads to significant tumor control, which depends on DCs and T cells (Martin et al., 2014). Herein, we show that MC38 tumors grow faster in GEF-H1^−/−^ mice compared with WT mice, although no significant differences in survival to endpoint were observed. In addition, the significantly larger tumors observed in ansamitocin-P3-treated GEF-H1^−/−^ compared with WT mice suggests that GEF-H1 regulates the anti-tumor efficacy of ansamitocin-P3 (Figure 6H).

Given the indication of a direct role of GEF-H1 in anti-tumor immune responses, we used The Cancer Genome Atlas (TCGA) to investigate the prognostic relevance in cancer patients of the proinflammatory GEF-H1-dependent immune signature obtained from Figure 3 (and outlined in Table S5). In at least three tumor types—melanoma, head and neck cancer, and uterine cancer—increased expression of the GEF-H1-dependent genes was associated with better overall survival (Figure 6I; Figures S6C and S6D). In addition, increased CD8A expression was noted in patients with higher expression of the GEF-H1 immune gene signature (Figure S6E). This suggests that the GEF-H1-dependent proinflammatory gene signature induced upon microtubule destabilization in DCs maybe prognostic, because it correlated with improved intratumoral T cell infiltration. Collectively, our findings indicated that GEF-H1 plays a critical role in initiating anti-tumor immunity, particularly upon treatment with MDAs such as ansamitocin-P3, and establishes a framework to guide the development of microtubule-targeting strategies.

## DISCUSSION

Here we demonstrate that GEF-H1 is essential for the induction of an innate immune activation pathway upon treatment with microtubule-targeting chemotherapy that can restore anti-tumor immunosurveillance. Upon destabilization of microtubules, GEFH1 is responsible for cell-intrinsic immune activation that leads to DC differentiation to cDCs with the ability to process and present antigens, as well as activate T cells. The specificity of the GEF-H1 pathway for DC activation is reserved for chemotherapies that destabilize microtubules (e.g., ansamitocin-P3, colchicine, and vinca alkaloids) and is not used for microtubule-stabilizing chemotherapies (e.g., paclitaxel and docetaxel).

Microtubules are highly dynamic cytoskeletal filamentous polymers composed of αβ-tubulin heterodimers and are the cellular targets of numerous chemotherapy drugs that either stabilize or destabilize microtubules (Jordan and Wilson, 2004). The latter typically bind to the vinca site (vinblastine, eribulin, and MMAE), to the colchicine site (colchicine, nocodazole, and plinabulin), or to the maytansine site on tubulin (ansamitocin-P3 and DM1) (Gigant et al., 2005; Ravelli et al., 2004; Prota et al., 2014; Steinmetz and Prota, 2018). Drugs with microtubule-destabilizing activity dominate the payloads within ADCs; most ADCs in clinical trials are conjugated to MMAE, monomethyl auristatin F (MMAF), DM1, or DM4 (Beck et al., 2017). Non-targeted novel microtubule-destabilizing drugs such as plinabulin have demonstrated durable clinical responses (Mohanlal et al., 2016). In addition to their tumor cytotoxicity, drugs altering microtubule dynamics are known to improve DC function (Mizumoto et al., 2005; Martin et al., 2014). Although such DC stimulatory effects are reserved for drugs with microtubule-destabilizing activity irrespective of their distinct tubulin-binding sites, intrinsic parameters such as cell permeability, compound stability, and expression of drug efflux pumps (Dumontet and Jordan, 2010) may influence their DC activation capacity.

Here, we demonstrate that a GEF-H1 variant comprising the C1, PH, and coiled-coil domains binds directly to microtubules, which upon action of MDAs on microtubules, is expected to be released and activated to induce DC maturational changes. In addition to microtubule-targeting drugs, anthracycline and its derivatives are known to promote DC maturation (Zitvogel et al., 2013). Anthracycline chemotherapies induce an immunogenic cell death (ICD) program in tumor cells, including the release of damage-associated molecular patterns (DAMPs), which are subsequently sensed by complementary PRRs, especially TLR4 expressed on DCs (Zitvogel et al., 2013). Anti-tumor immunity observed with anthracycline chemotherapy is mechanistically distinct from the microtubule-destabilizing chemotherapy reported herein. The latter is primarily mediated through its direct action on DCs and thus employs alternate mechanisms distinct from ICD. We observed no significant impact of the lack of TLR4, TRIF, or NALP3 on DC maturational changes upon microtubule destabilization. Upregulation of CD40, CD86, and MHC class II occurred independently of MyD88, a cytosolic adaptor protein shared by most TLRs (Müller et al., 2014a). However, the intracellular GEF-H1 signaling was critical in initiating DC maturation upon microtubule destabilization and induction of immune responses such as proinflammatory cytokine production (e.g., IL-1, IL-6, and IL-12) that otherwise require extracellular and intracellular microbial pattern recognition. These findings are in agreement with a specific function of GEF-H1 in microtubule-dependent signaling of intracellular nucleic acid detection pathways, while extracellular pattern recognition through TLRs occurs independent of microtubules (Chiang et al., 2014).

In line with our finding and in contrast to the critical role of PRRs in mediating immunological responses to anthracycline chemotherapies, mice deficient in TLR or IL-1 receptor signaling display no defect in spontaneous or radiation-induced T cell responses against tumors (Deng et al., 2014; Woo et al., 2014). These findings suggest an alternate pathway leading to effective DC activation, which may be advantageous to engage, particularly in the tumor microenvironment. In addition, the activation of IRF5 and NF-κB suggests that the MDAs investigated here can initiate a GEF-H1-dependent innate immune pathway that is activated in response to microbial peptidoglycans (Zhao et al., 2019).

Though agonists of PRRs are in clinical development mainly as adjuncts to cancer immunotherapy strategies (Shekarian et al., 2017), chronic activation of TLRs may induce protumorigenic effects (Pandey et al., 2015). Furthermore, PRR expression is specific for distinct DC subsets, which results in variable responsiveness to PRR targeting depending on DC infiltration profiles (Gilliet et al., 2008). Hence, careful investigation of alternate pathways that lead to DC activation and effective anti-tumor immunity such as the ones proposed herein are of high relevance in the landscape of immune oncology.

We used RNA-seq to better characterize the intracellular signaling pathways and transcriptional responses upon microtubule destabilization in DCs. RNA-seq analyses revealed the extent and specificity of the GEF-H1-dependent immune response in DCs in the context of microtubule destabilization. Gene enrichment analysis associated the regulated gene clusters with inflammatory signaling and the control of adaptive T cell-mediated immune responses. The involvement of the AP-1 transcription family, particularly c-Jun, in the treatment response was independently identified in our gene expression analyses, unbiased coexpression analyses, and protein phosphorylation or activation experiments. c-Jun is part of the dimeric transcription factor AP-1 complexes that assemble from members of the Jun (c-Jun, JunB, and JunD), Fos (c-Fos, FosB, Fra-1, and Fra-2), ATF, and MAF protein families (Karin et al., 1997). Its upstream signaling regulators, namely, RhoA, MKK4, and JNK1/2, were seen in our study to feed into the AP-1 transcriptional response in a GEF-H1-dependent manner. Although AP-1 activation is also a hallmark for pathogen recognition pathways, DC activation upon treatment with microtubule-destabilizing chemotherapy was independent of PRRs. The SRF transcription factor (TF) motif, the highest enriched gene set in our GSEAs, is regulated by the Rho family GTPases, including RhoA, Rac, and Cdc42 (Hill et al., 1995), which are downstream substrates of GEF-H1. This is known to affect cytoskeletal dynamics, including actin, which may alter antigen processing and T cell priming.

However, animals lacking GEF-H1 signaling were unable to efficiently cross-present antigens to CD8 T cells upon microtubule destabilization and consequently were more refractory to therapy-induced anti-tumor immunity. This is surprising, because GEF-H1 is implicated in the differentiation of DCs in the Trif-GEF-H1-RhoB pathway involved in MHC class II expression (Kamon et al., 2006). Because MHC class I-specific OVA_257–264_ peptide presentation was not impaired in GEF-H1^−/−^ DCs, the precise mechanism by which GEF-H1 controls antigen processing in DCs will need to be further investigated. Nevertheless, there is evidence for the role of GEF-H1 in membrane trafficking and recycling (Arnette et al., 2016), wherein the loss of GEF-H1 impaired recycling endosomes and the post-Golgi secretory vesicles (Ullrich et al., 1996). This indicates that the intracellular machinery used for antigen cross-presentation upon microtubule destabilization is hampered in the absence of GEF-H1. Altered CD8 T cell expansion after full-length OVA immunization, but not after OVA peptide immunization, indicates that GEF-H1^−/−^ DCs have impaired intracellular antigen processing capabilities that are required for cross-presentation.

The more rapid growth of untreated MC38 tumors in GEFH1^−/−^ animals in the early phase of tumor immune control, i.e., when the tumor burden is low, indicates that the GEF-H1 axis may be involved in the early events that control tumor immunity, DC activation, and tumor antigen presentation. Thus, microtubule-based control mechanisms may exist that naturally govern DC maturation that are amplified by MDAs. The clinical relevance of the GEF-H1 immune pathway is supported by our TCGA analysis, which shows a significant association of CD8A to the GEF-H1 immune gene signature in patients with melanoma, head and neck cancer, and uterine cancer. This suggests that tumors with active GEF-H1 signaling have improved anti-tumor immunity, resulting in decreased risk of death. Better definition of the predictive potential of this pathway would require a TCGA dataset from patients treated with microtubule-destabilizing chemotherapy. In addition, because selection criteria for patient data available in TCGA are unknown, it is not possible to account for potential confounding factors that may have biased this analysis using standard statistical analysis techniques (McShane et al., 2005). Our findings identify GEF-H1-dependent immune activation events in DCs that could be harnessed for the design of immunotherapy approaches extending beyond microtubule-targeting chemotherapy. For instance, radiotherapy, which is exceedingly being used and combined with immunotherapy (Marciscano et al., 2018), is known to influence tubulin content and cause microtubule destabilization (Zaremba and Irwin, 1981; Woloschak et al., 1990), which may thereby directly activate GEF-H1 to boost DC function.

In summary, we demonstrate that an alternate cell-intrinsic pathway of DC maturation is induced upon microtubule destabilization by GEF-H1 that is capable of reinstating and enhancing anti-tumor immune responses. DC activation by the GEF-H1 pathway may be used to overcome the immune tolerant tumor environment and improve the utility of current immune checkpoint blockade and personalized cancer vaccinations.

## STAR★METHODS

### LEAD CONTACT AND MATERIALS AVAILABILITY

Further information and requests for resources and reagents should be directed to and will be fulfilled by the Lead Contact, Alfred Zippelius (alfred.zippelius@usb.ch).

Plasmids (GEFH1-C1-PH-GCN4 and GEFH1 sgRNA-pSpCas9(BB)-2A-GFP) and mouse cell lines (XS106 GEFH1^−/−^) generated in this study will be made available on request but we may require a completed Materials Transfer Agreement.

### EXPERIMENTAL MODEL AND SUBJECT DETAILS

#### Animals

C57BL/6N wild-type, OT-I and OT-II TCR transgenic mice were bred in-house either at University Hospital Basel, Switzerland or Massachusetts General Hospital (MGH), USA. In case of unavailability mice were also obtained from Jackson Laboratories (USA) or Janvier Labs (France). GEFH1^−/−^ mice on C57BL/6N background were generated as previously published (Chiang et al., 2014) and were bred at MGH. 129S.Zbtb46-GFP reporter mice (obtained from Jackson Laboratories) were also bred at MGH. All animals were bred and housed in a pathogen-free animal facility according to institutional guidelines. All experiments were carried out on sex-matched mice at 8–16 weeks old, both males and females were used with no influence on results. All animals were maintained under a strict 12 h light cycle (lights on at 5:00 a.m. and off at 5:00 p.m.), and given food and water available *ad libitum*. All animal experiments were performed in accordance with Swiss federal regulations at University Hospital Basel (Basel Kantonal license numbers: 2370, 2589 and 2408) and the Subcommittee of Research Animal Care at at the Massachusetts General Hospital and Harvard Medical School (protocol number 2011N000089).

#### Cell Lines

COS-7 fibroblast cells were purchased from American Type Culture Collection (ATCC), maintained in DMEM supplemented with 10% fetal bovine serum (FBS) and 0.5% penicillin-streptomycin (P/S; GIBCO) mixture. The immature mouse DC cell line SP37A3 (kindly provided by Merck KGaA) was cultured in Iscove’s Modified Dulbecco’s Medium (IMDM; Sigma) supplemented with 10% heat-inactivated FBS (PAA), sodium pyruvate (GIBCO), P/S, L-glutamine mix (GIBCO), MEM nonessential amino acids (Sigma), and with 20 ng/mL recombinant mouse GM-CSF and 20 ng/mL recombinant mouse M-CSF (both Peprotech). XS106 cell line (kind gift from Professor Akira Takashima, University of Texas South-Western, TX, USA) is a long-established DC line derived from the epidermis of newborn mice 56 and are better suited for lipid/viral transfection compared to SP37A3 cells. These cells were cultured in RPMI-1640 medium supplemented with 10% FBS and 0.5% P/S. The medium was further supplemented with 20 ng/mL murine recombinant GM-CSF and 5% (v/v) culture supernatant derived from the NS47 fibroblast cell line. The NS-47 cell line was cultured in RPMI-1640 complete medium. All cells were cultured at 37° in a 5% CO2/air atmosphere. GEFH1 deficient XS106 cells were created using CRISPR/Cas9 mediated gene editing. Two guide RNAs (GCACATGGTCATGCCGGAGA and GACAAGGTAGGAGTCAGCCT) were designed using the online tool e-crisp.org, synthesized by Microsynth (Switzerland) and cloned into the pSpCas9(BB)-2A-GFP (PX458) vector (Addgene plasmid #48138). After transient transfection, XS106 cells were single cell sorted according to GFP expression, expanded and subsequently screened for GEFH1 expression by western blot.

#### Primary Cell Culture

Bone marrow derived DCs were generated by plating 5 million bone marrow cells freshly isolated from tibia and femur of C57BL/6N mice into 10 cm dishes. RPMI-1640 supplemented with 10% heat inactivated FCS, 0.5% P/S, GM-CSF (10 ng/mL; Peprotech) and IL-4 (10 ng/mL; Peprotech) was used to culture the BM cells. On day 6, floating and loosely attached cells were collected representing the BMDCs. Briefly, spleens were collected and cut into fine pieces and digested with Collagenase type D (1 mg/ml, Roche) and DNase I (40 mg/ml, Roche) in RPMI 10% FCS for 40 minutes at 37°C. Single cell suspensions were obtained by passing the digested tissue through a 70 mm strainer using ice-cold PBS supplemented with 0.5 mM EDTA and 2% FCS. The DCs were isolated by immunomagnetic CD11c+ positive selection according to manufacturer’s protocol (StemCell Technologies). The purity of the splenic DCs was also assessed by flow cytometry and was typically between 80%–90%.

### METHOD DETAILS

#### Reagents and Antibodies

Anti-cancer agents namely, ansamitocin-P3 (Cayman Chemicals), plinabulin (kindly provided by BeyondSpring Pharmaceuticals), eribulin (kindly provided by Eisai Co. Ltd), MMAE (kindly provided by Seattle Genetics), DM1 (Concortis Biosystems), colchicine (Sigma Aldrich), vinblastine (National Cancer Institute), nocodazole (Sigma Aldrich), dolastatin-10 (National Cancer Institute), epothilone-A (Santa Cruz Biotechnology), docetaxel (Selleckchem), paclitaxel (Cayman Chemicals), CW190 (Prof. Altmnann, ETH Zurich) and etoposide (Sigma Aldrich) were dissolved in 100% DMSO (10 mM stock) and tested at various concentrations with a final maximum DMSO concentration of 0.1%. Endotoxin-free ovalbumin (OVA) protein (EndoFit) was purchased from InvivoGen. Lipopolysaccharide (LPS) from *Escherichia coli* 0111:B4 was purchased from InvivoGen. The following antibodies for immunoblotting were obtained from Cell Signaling: phospho JNK (81E11), JNK, phospho p65-NFκB (93H1), p65-NFκB (D14E12), phospho ERK1/2 (D13.14.4E), ERK1/2 (137F5), phospho p38-MAPK (12F10), p38 MAPK (D13E1), phospho MKK4 (C36C11), MKK4, phospho MKK3 (D8E9), MKK3 (D4C3), phospho c-Jun (D47G9), c-Jun (60A8), phospho IRF3 (4D4G), IRF3 (D83B9), IRF5 phospho STAT1 (58D6), STAT1 (cat no. 9172), and β-actin (8H10D10). Antibodies for phospho GEFH1 (ab74156), anti-IRF5 (ab21689) and αTubulin were purchased from Abcam. Anti-GEFH1 antibody (x1089p) was purchased from Exalpha Biologicals. The anti-IRF5 phosphorylated at Ser 445 was produced by NeoBiolab (MA, USA) by immunizing rabbits with a synthetic peptide (IRLQIPS^445^NPDLC). Plasmids encoding GFP-GEFH1 (pCMV6-AC-GFP-hGEFH1) were purchased from OriGene.

#### Stimulation of Murine DCs *In Vitro*

Pre-seeded day 6 BMDCs (80,000 cells/well of 96-well plate), freshly isolated splenic DCs (160,000 cells/well of 96-well plate), murine SP37A3 DC cells or murine XS106 DC cells (80,000 cells/well of 96-well plate) were incubated with microtubule targeting agents or LPS at the indicated concentrations. After 20 hours, unless otherwise stated, the DCs were harvested using PBS/EDTA detachment and their phenotype was assessed either by flow cytometry or ELISA.

#### Measurement of Cytokine Production

IL-1β, IL-6, and IL-12 in supernatants of murine DC cultures pre- and post-stimulation were detected by standard sandwich ELISA procedures using commercially available kits (eBioscience) following manufacturer’s instructions.

#### Analyses of mRNA Expression

Murine BMDCs were isolated and treated as described above. QIAGEN RNeasy kit was used for the extraction of RNA. cDNA was synthesized using the iScript cDNA synthesis kit (Bio-Rad) following which SsoAdvanced Universal SYBR Green supermix kit (Bio-Rad) was used for real-time qPCR (Bio-Rad CFX96 Real-Time PCR Detection System) according to the manufacturer’s specifications. The value obtained for each gene was normalized to that of the *GAPDH* gene. Primers used were as follows (all 5′ to 3′). Il1b-F: GCAACTGTTCCTGAACTCAACT, IL1b-R: ATCTTTTGGGGTCCGTCAACT; Il6-F: CCTAGTTGTGATTCTTTCGATGCT, Il6-R: ACAGACATCCCCAGTCTCATATTT; Il12a-F: AGACATCACACGGGACCAAAC, Il12a-R: CCAGGCAACTCTCGTTCTTGT; IL12b-F: TGGTTTGCCATCGTTTTGCTG, IL12b-R: ACAGGTGAGGTTCACTGTTTCT; CD80-F: TCGTCTTTCACAAGTGTCTTCAG, CD80-R: TTGCCAGTAGATTCGGTCTTC; CD86-F: GAAGCCGAATCAGCCTAGC, CD86-R: CAGCGTTACTATCCCGCTCT; Gapdh-F: TGACCTCAACTACATGGTCTACA, Gapdh-R: CTTCCCATTCTCGGCCTTG.

#### Immunoprecipitation and Immunoblotting

To assess phosphorylated and total GEFH1, day 6 BMDCs treated with ansamitocin-P3 (100 nM) or taxane (100 nM) at indicated time points were lysed using NP-40 buffer (1% NP-40, 20 mM Tris-HCl at pH 7.4, 150 mM NaCl, 2 mM EDTA, 2 mM EGTA, 4 mM Na_3_VO_4_, 40 mM NaF) containing protease and phosphatase inhibitors (Complete Mini tablet; Roche). Lysates were used for direct assessment by western blotting or for GEFH1 immunoprecipitation. For immunoprecipitation, lysates were incubated with protein G plus agarose (Calbiochem) at 4°C for 30 minutes and pre-cleared. Pre-cleared lysates were incubated with anti-GEFH1 antibody (1:200) at 4°C overnight followed by incubation with agarose beads at 4°C for 4 hours. Precipitated proteins were collected by centrifugation and washed 3 times in washing buffer (0.5% NP-40, 20 mM Tris-HCl at pH 7.4, 150 mM NaCl, 2 mM EDTA, 2 mM EGTA, 4 mM Na_3_VO_4_, 40 mM NaF). After washing, proteins were boiled with SDS-PAGE sample buffer at 95°C for 10 minutes and detected by western blotting. Membranes were blocked with 5% non-fat dry milk in Tris-buffered saline (TBS) at room temperature for 1 hour and incubated with primary antibodies against the phosphorylated protein diluted in blocking solution to a ratio of 1:1000 at 4°C overnight. After washing in TBS with 0.05% Tween-20 (TBS-T), membranes were incubated with appropriate horseradish peroxidase conjugated secondary antibody diluted in blocking buffer for 1 hour at room temperature. Blots were washed 3 times with TBS-T and hybridized bands were detected by Amersham ECL western blotting detection reagent (GE Healthcare). The blots probed for the phosphorylated proteins were stripped and re-probed with antibodies for the respective total proteins.

#### Confocal Live Cell Imaging

COS-7 fibroblasts pre-seeded into 4-well chamber slides (LabTek) were transfected with 1 μg of the GFP-GEFH1 plasmid using Lipofectamine 3000. Live cells were imaged 20 hours post transfection with a Nikon A1R-A1 confocal microscope. Images were acquired immediately upon the addition of ansamitocin-P3 (1 μM) or taxane (1 μM). Image acquisition was carried out with NIS-Elements imaging software (Nikon) followed by analyses by Volocity (PerkinElmer).

#### Cloning and Production of GEFH1 Constructs

The human GEFH1 (Uniprot Q92974–1) C1 (residues 28–100) and PH domains (residues 439–589) were initially cloned in isolation into a pET-based bacterial expression vector containing an N-terminal thioredoxin-6xHis cleavable tag using a restriction free positive selection method (Olieric et al., 2010). The GEFH1-C1-PH-GCN4 construct was assembled by homologous recombination using overlapping PCR fragments by fusing in frame the leucine zipper coiled-coil domain of the yeast transcriptional activator GCN4 (O’Shea et al., 1991) C-terminally to the PH domain. All clones were verified by sequencing.

Protein samples were produced by overexpression in *E. coli* Bl21(DE3) cells. Protein purification was performed by immobilized metal-affinity chromatography (IMAC) on HisTrap HP Ni^2+^ Sepharose columns (GE Healthcare) according to the manufacturer’s instructions. Processed protein samples were concentrated and processed on a HiLoad Superdex 200 16/60 size exclusion chromatography column (GE Healthcare) equilibrated in 50 mM Tris HCl, pH 7.5, supplemented with 150 mM NaCl and 2 mM DTT. Protein fractions were analyzed by Coomasie stained SDS-PAGE. Fractions containing the target protein were pooled and concentrated by ultrafiltration. Protein concentrations were estimated by UV absorbance at 280 nm.

#### *In vitro* Microtubule Pelleting Assay

Microtubule binding of GEFH1 variants was performed by a standard microtubule co-sedimentation assay (Devred et al., 2010). Briefly, tubulin at 2 mg/mL in BRB80 buffer (80 mM PIPES-KOH, pH 6.8, 1 mM MgCl_2_, 1 mM EGTA) supplemented with 0.5 mM GTP and 1.25 mM DTT was incubated at 4°C for 5 minutes followed by incubation at 37°C for 10 minutes. Taxol was added to the reaction mix in a step wise manner (0.1, 1, and 10 μM) to induce microtubule formation. Taxol-stabilized microtubules were mixed with test proteins (ranging from 0.125 to 2 mg/mL). The reaction mixture was added on top of a Taxol-glycerol cushion (2X BRB80, 40% glycerol, 20 mM taxane). After high-speed centrifugation (80,000 rpm, 30 min, 30°C), the microtubule-rich pellet fraction was separated from the supernatant fraction. Each fraction was analyzed on 12% SDS-PAGE followed by Coomasie staining.

#### Flow Cytometry

Flow cytometry was performed on cell lines, BMDCs or cells isolated from spleen, lymph nodes or skin. Single cell suspensions were washed with PBS and stained with the fixable live/dead UV Zombie dye (BioLegend). Cells were then blocked with Fc receptor-blocking anti-CD16/32 antibody (clone 2.4G2; 1:100) for 20 minutes at 4°C and stained for cell surface antigens using the following fluorophore-conjugated anti-murine antibodies for 20 minutes at 4°C: CD11c-PE-Cy7 (clone HL3; 1:200), MHCII-BV510 (clone M5/144.15.2; 1:200), CD11b-APC-Cy7 (clone M1/70; 1:200), CD86-APC (clone GL-1; 1:300), CD80-PE (clone 16–10A1; 1:300), CD45-APC-Cy7 (clone 30-F11; 1:300), CD40-BV421 (clone 3/23; 1:200), TCRVb5-APC (clone MR9–4; 1:200). Washing and antibody incubations were performed in FACS buffer (PBS, 0.5 mM EDTA, 2% FCS). Cells were either fixed with IC fix buffer (eBioscience) for 20 minutes or were directly acquired on LSR Fortessa or FACS Aria III (both BD Bioscience).

#### *In vitro* Stimulation of OVA-Specific OT-I and OT-II T Cells

SP37A3 cells or day 6 BMDCs were pulsed for 1 hour with OVA full-length protein (0.1 mg/mL) before activation with ansamitocin-P3 (100 nM), taxane (100 nM) or LPS (100 ng/mL) and added at the indicated ratios to CD8 or CD4 T cells purified (by magnetic selection; Miltenyi Biotec) from spleen and LN of OT-I/OT-II transgenic mice (2 ×10^5^ total cells/well, 96-well round bottomed plate). The CD8 and CD4 T cells were loaded with the proliferation dye CellTrace Violet (Molecular Probes) before co-culture following manufacturer’s instructions. Proliferation was assessed after 3 days using flow cytometry.

#### *In vivo* Activation of Skin DCs

Ansamitocin-P3 (4 μg/ear) or LPS (8 μg/ear) or Vehicle (1.5% DMSO) was injected intradermally into the ears of C57BL/6N WT or GEFH1^−/−^ mice. Analysis was performed after 24 hours using flow cytometry. Epidermal sheets were digested with Accutase (Sigma), collagenase IV (Worthington), hyaluronidase (Sigma), and DNase type IV (Sigma). Single-cell suspensions were prepared and stained with anti-CD45, anti-CD11c, anti-MHC-II, anti-CD86 and anti-CD80 antibodies. Dead cells were excluded using Zombie UV dye (BioLegend).

#### *In Vivo* Stimulation of Antigen-Specific CD8 and CD4 T Cells

CD8 and CD4 T cells from LNs and spleen of naive OT-I and OT-II transgenic mice, respectively, were purified using magnetic separation (Miltenyi Biotec) and labeled with CellTrace Violet (Molecular Probes) following manufacturer’s instructions. Two million CD8 or CD4 T cells were adoptively transferred i.v. into C57BL/6N WT or GEFH1^−/−^ mice. After 24 hours, mice were immunized via tail-base injection with full length OVA protein (25 μg/mouse) together with ansamitocin-P3 (4 μg/mouse) or LPS (25 μg/mouse) or vehicle(0.5% DMSO). Three days after immunization draining lymph nodes (iliac, axial and inguinal) were collected and proliferation of the adoptively transferred OT-I CD8 and OT-II CD4 T cells was assessed by flow cytometry.

#### *In Vivo* Tumor Challenge and Treatment Protocol

C57BL/6N WT or C57BL/6N GEFH1^−/−^ mice were injected subcutaneously into the right flank with 500,000 syngeneic MC38 cells suspended in phenol red-free DMEM (without additives). Mice bearing palpable MC38 tumors received peri-tumoral injection of 50 μL ansamitocin-P3 (0.3 mg/kg) or vehicle (2% DMSO) on days 8, 9 and 10 post tumor challenge. Tumor volume was calculated according to the formula: D /2*d*d, with D and d being the longest and shortest tumor diameter in mm, respectively.

#### RNaseq and GSEA Analyses

RNA was isolated from C57BL/6N WT and GEFH1^−/−^ DCs using RNeasy Micro kit (QIAGEN) following the manufacturer’s instructions. Libraries were synthesized using Illumina TruSeq Stranded mRNA sample preparation kit from 500 ng of purified total RNA and indexed adaptors according to the manufacturer’s protocol (Illumina). The final dsDNA libraries were quantified by Qubit fluorometer, Agilent Tapestation 2200, and RT-qPCR using the Kapa Biosystems library quantification kit according to manufacturer’s protocols. Pooled libraries were subjected to 35-bp paired-end sequencing according to the manufacturer’s protocol (Illumina Next-Seq 500). Targeted sequencing depth was 25 million paired-end reads per sample. Blc2fastq2 Conversion software (Illumina) was used to generate de-multiplexed Fastq files.

Expression values were normalized as Fragments per Kilobase Million reads after correction for gene length (FPKM) in Cuffdiff version 1.05 in the DNAnexus analysis pipleline. We filtered for statistically significant (p < 0.01) genes with a false discovery rate (FDR) threshold of 0.05 and a biologically relevant change (log fold change > 1; logFC). Samples were analyzed in the RNasequencing pipeline of Seqmonk for mRNAs for opposing strand specific and paired end libraries with merged transcriptome isoforms, correction for DNA contamination and log transformed resulting expression values in log2FPM. Ansamitocin-P3 induced mRNAs that were differentially regulated more that 2-fold (FDR threshold of 0.05) in the Cuffdiff analysis of WT DCs were imported into Seqmonk for per-probe normalized hierarchical clustering of mRNA transcription in control and ansamitocin-P3 stimulated WT and GEFH1 deficient DCs.

To generate a ranked gene list for GSEA analyses stranded reads were aligned and counted using STAR (2.5.2a) (Dobin et al., 2013) in stranded union mode using Illumina’s ENSEMBL iGenomes GRCm38 build and GRCm38.90 known gene annotations. Count level data was then analyzed using the edgeR Bioconductor package in R (Robinson et al., 2010). Filtered genes, expressed at > 1 count per million (cpm) in at least two samples, were analyzed using the QLF functions comparing WT and GEFH1^−/−^ BMDCs untreated and ansamitocin-P3-treated samples. All genes were ranked according to their –log10 transformed corrected p value for differential up/downregulation by ansamitocin-P3 in WT versus GEFH1^−/−^ BMDCs. Mouse genes were mapped to their human orthologs using HCOP (http://www.genenames.org/cgi-bin/hcop at 8.9.17). The pre-ranked list was used to perform weighted GSEA using the GSEA java application (http://software.broadinstitute.org/gsea/index.jsp) that uses the Molecular Signature Database (MSigDB).

#### Co-expression Enrichment Analysis

Co-expression analysis interrogates mouse co-expression maps generated by collecting 3571 microarray datasets irrespective of treatment conditions and tissues (van Dam et al., 2012). The co-expression map highlights the co-expression patterns without enrichment for particular tissue or condition among the datasets. The genes that are dependent on both the treatment, and GEFH1 were used for the co-expression enrichment analysis (clusters I and III from Figure 3C; 831 genes). This gene signature was used as the input to the online tool (http://www.genefriends.org) that produced a ranked list of genes co-expressed with the signature. This tool restitutes the full list of mouse genes (22,766 genes) ordered by the connectivity score to our GEFH1-dependent gene list. From the full list (22,766 genes) we extracted our 831 genes that were then ordered by their interconnectivity within the gene list itself. From this list we took the top 80 co-expressed genes that also belonged within our gene signature. This procedure allowed us to select in an unbiased manner the genes that have a central role within the gene signature matrix. Among the co-expressed genes, we reported the top 15 transcription factors, which are then very likely to be the main drivers of the expression of our GEFH1-related signature. The analysis was repeated using the human orthologs and interrogating the human co-expression network (Monaco et al., 2015). The R package ComplexHeatmap was used to generate the heatmap of the gene expression of the selected 80 genes.

#### Integrated System for Motif Activity Response Analysis (ISMARA)

Unprocessed read data in fastq format was submitted for ISMARA analysis through the https://ismara.unibas.ch/ online platform for RNASeq using the mm10 assembly settings as described (Balwierz et al., 2014). Conditions were averaged and the most significantly changed motif activities were extracted (z-score).

#### Analysis of TCGA Datasets

From the differential expression analysis described in the previous section, we selected the genes that were upregulated upon ansamitocin-P3 treatment and dependent to GEFH1 (FDR < 0.05 and Fold Change > 2). Immune specific genes were extracted using the LM22 matrix (Newman et al., 2015) to deconvolute immune signals from tumor samples. RNA-seq datasets of all solid tumors of the TCGA database were downloaded with the R package TCGAbiolinks (Colaprico et al., 2016). For all patients the FPKM value of each gene within the GEFH1 immune signature was log2 transformed and the median expression of the gene signature was used as a surrogate marker of GEFH1 activity. We used univariable Cox regression analyses to investigate the association between the median expression of the gene signature (continuous independent variable) and survival (dependent variable). To account for possible non-linear associations and to circumvent choosing arbitrary cut-points, we used the multivariable fractional polynomial approach (Sauerbrei et al., 2007) for the Cox model. By qualitative assessment of the resulting regression plots, we identified a cut-off at 14 as clinically important and created Kaplan-Meier plots to visualize the difference in survival. To investigate the association between the gene signature and survival across several tumor types, we used techniques of random and fixed effects meta-analysis. Hazard ratios from each Cox regression model (by each tumor type) were pooled; results from this prognostic meta-analysis are visualized by a forest plot. Associations are expressed with hazard ratios accompanied by 95% confidence intervals.

### QUANTIFICATION AND STATISTICAL ANALYSIS

All samples or animals from each experiment were included for analysis. GraphPad Prism was used for all statistical analysis. Statistical analysis was carried out by two-way analysis of variance (ANOVA) followed by Tukey’s post hoc test for grouped analyses or by one way ANOVA followed by Tukey’s test in case of non-grouped analyses. p < 0.05 was considered statistically significant. All graph bars included mean and standard deviation to depict the error.

### DATA AND CODE AVAILABILITY

The RNaseq data supporting the findings of this study are available within the paper and its Supplemental Information files. The raw FASTQ files are deposited in NCBI GEO under accession number GSE135264.

## Supplementary Material

1

2

3

4

5

6

7

8

## Figures and Tables

**Table:** KEY RESOURCES TABLE

REAGENT or RESOURCE	SOURCE	IDENTIFIER
Antibodies		
Rabbit monoclonal phospho JNK (Thr183/Tyrl85) (81E11)	Cell Signaling	Cat# 4668
Rabbit Anti-Mouse JNK	Cell Signaling	Cat# 9252
Rabbit Anti-Mouse phospho p65-NFκB (93H1)	Cell Signaling	Cat# 3033
Rabbit Anti-Mouse p65-NF_K_B (D14E12)	Cell Signaling	Cat# 8242
Rabbit Anti-Mouse phospho ERK1/2 (D13.14.4E)	Cell Signaling	Cat# 4370
Rabbit Anti-Mouse ERK1/2 (137F5)	Cell Signaling	Cat# 4695
Rabbit Anti-Mouse phospho p38-MAPK (12F10)	Cell Signaling	Cat# 4511
Rabbit Anti-Mouse p38 MAPK (D13E1)	Cell Signaling	Cat# 8690
Rabbit Anti-Mouse phospho MKK4 (C36C11)	Cell Signaling	Cat#4514
Rabbit Anti-Mouse MKK4	Cell Signaling	Cat#9152
Rabbit Anti-Mouse phospho MKK3 (D8E9)	Cell Signaling	Cat# 12280
Rabbit Anti-Mouse MKK3 (D4C3)	Cell Signaling	Cat# 8535
Rabbit Anti-Mouse phospho c-Jun (D47G9)	Cell Signaling	Cat# 3270
Rabbit Anti-Mouse c-Jun (60A8)	Cell Signaling	Cat#9165
Rabbit Anti-Mouse phospho IRF3 (4D4G)	Cell Signaling	Cat# 4947
Rabbit Anti-Mouse IRF3 (D83B9)	Cell Signaling	Cat# 4302
Rabbit Anti-Mouse phospho STAT1 (58D6)	Cell Signaling	Cat#9167
Rabbit Anti-Mouse STAT1	Cell Signaling	Cat#9172
Mouse anti-β-actin (8H10D10)	Cell Signaling	Cat# 3700
Rabbit Anti-phospho GEFH1	Abcam	Cat# ab74156
Rabbit Anti-IRF5	Abcam	Cat# ab21689
Rabbit Anti-alpha Tubulin	Abcam	Cat# ab15246
Sheep Anti-Mouse GEFH1 antibody	Exalpha Biologicals	Cat# X1089P
Anti-phospho IRF5 (Ser-445)	NeoBiolab (MA, USA)	N/A
Zombie UV Fixable Viability Kit	BioLegend	Cat# 423107
Anti-Mouse TCRVb5-APC (clone MR9–4) (1:200 dilution)	BioLegend	Cat# 139505
Anti-Mouse MHCII (l-A/l-E)-BV510 (clone M5/144.15.2) (1:200 dilution)	BioLegend	Cat# 107636
Anti-Mouse CD11b-APC-Cy7 (clone M1/70) (1:200 dilution)	BioLegend	Cat# 101226
Anti-Mouse CD86-APC (clone GL-1) (1:300 dilution)	BioLegend	Cat# 105012
Anti-Mouse CD80-PE (clone 16–10A1) (1:300 dilution)	BioLegend	Cat# 104707
Anti-Mouse CD45-APC-Cy7 (clone 30-F11) (1:300 dilution)	BioLegend	Cat# 103116
Anti-Mouse CD40-BV421 (clone 3/23) (1:200 dilution)	BD Biosciences	Cat# 562846
Anti-Mouse CD11-c-Pe-Cy7 (clone HL3) (1:200 dilution)	BD Biosciences	Cat# 561022
Bacterial and Virus Strains		
pSpCas9(BB)-2A-GFP (PX458) vector	Ran et al., 2013	Addgene Plasmid; Cat# 48138
GEFH1-C1-PH-GCN4 construct	This paper	N/A
GEFH1 sgRNA-pSpCas9(BB)-2A-GFP	This paper	N/A
Chemicals, Peptides, and Recombinant Proteins		
Ansamitocin-P3	Cayman chemicals	Cat# 20538
Dolastatin-10	National Cancer Institute	N/A
Vinblastine	National Cancer Institute	N/A
Colchicine	Sigma Aldrich	Cat# C9754
Nocodazole	Sigma Aldrich	Cat# M1404
Etoposide	Sigma Aldrich	CAS: 33419-42-0
Hyaluronidase	Sigma-Aldrich	Cat# H6354
DNase type IV	Sigma-Aldrich	Cat# D5025; CAS: 9003-98-9
Dimethyl sulfoxide (DMSO)	Sigma-Aldrich	Cat# D2650; CAS: 67-68-5
Epothilone-A	Santa Cruz Biotechnology	Sc-207628; CAS: 152044-53-6
Docetaxel	Selleckchem	Cat#S1148
Paclitaxel	Cayman Chemicals	Cat# 10461; CAS: 33069-62-4
CW190	Prof. Altmnann, ETH Zurich	N/A
Accutase	Sigma Aldrich	A6964
EndoFit Endotoxin-free ovalbumin protein	InVivo Gen	vac-pova-100
Lipopolysaccharide from *Escherichia coli* 0111:B4	InVivo Gen	Ultrapure LPS, *E. coli* 0111:B4
Collagenase Type 4	Worthington	Cat#LS004189
CellTrace Violet	Molecular Probes	Cat# C34557
Phosphatase Inhibitor (PhosSTOP)	Roche	Cat# 4906845001
Protein G Plus/Protein A Agarose	Calbiochem	Cat#IP0414ML
SDS-PAGE sample buffer	Bio-Rad	Cat# 1610747
ECL Western Blotting Detection reagents	GE Healthcare	Cat# GERPN2209
Plinabulin	BeyondSpring Pharmaceuticals	N/A
Eribulin	Eisai Co. Ltd	N/A
MMAE	Seattle Genetics	N/A
DM1	Concortis Biosystems	N/A
Critical Commercial Assays		
EasySep Mouse CD11c Positive Selection Kit II	STEMCELL Technologies	Cat #18780
IL-1β Mouse ELISA kit	eBioscience	Cat# BMS6002
IL-6 Mouse ELISA kit	eBioscience	Cat# BMS603–2
IL-12 Mouse ELISA kit	eBioscience	Cat# BMS616
IC Fixation buffer	eBioscience	Cat# 00-8222-49
Mouse CD4+ T Cell Isolation Kit	Miltenyi Biotec	Cat# 130-104-454
Mouse CD8a+ T Cell Isolation Kit	Miltenyi Biotec	Cat# 130-104-075
RNeasy kit	QIAGEN	Cat#74104
iScript cDNA synthesis kit	Bio-Rad	Cat#1708890
SsoAdvanced Universal SYBR Green supermix kit	Bio-Rad	Cat# 172–5270
TruSeq Stranded mRNA sample preparation kit	Illumina	Cat# 20020594
Kapa Biosystems library quantification kit	Roche	N/A
Deposited Data		
Raw RNaseq data	This paper	GEO:GSE135264
Experimental Models: Cell Lines		
COS-7 fibroblasts cells	American Type Culture Collection (ATCC)	N/A
SP37A3 (immature dendritic cell line)	Merck KGaA	
XS106 cell line	Professor Akira Takashima, University of Texas, USA	N/A
NS47 fibroblast cell line	Professor Akira Takashima, University of Texas, USA	N/A
XS106 GEFH1^−/−^	This paper	N/A
E.coli Bl21 (DE3) cells	NEB Biolabs	Cat# C2527I
Experimental Models: Organisms/Strains		
Mouse: C57BL/6NRj wild type	In house	N/A
Mouse: OT-I (B6.129S6-*Rag2*^*tm1Fwa*^ Tg(TcraTcrb) 11OOMjb)	In house	N/A
Mouse: OT-II (B6.129S6 *Rag2*^*tm1Fwa*^ Tg(TcraTcrb) 425Cbn)	In house	N/A
Mouse: 129S.Zbtb46^tm1Kmm^/J	The Jackson Laboratories	Stock No: 000690
Mouse: GEFH1^−/−^(B6.Arhgef2 < tm1 Hcr >)	In house	N/A
Oligonucleotides		
Primer Il1b-Forward: GCAACTGTTCCTGAACTCAACT	Microsynth	N/A
Primer Il6-Forward: CCTAGTTGTGATTCTTTCGATGCT	Microsynth	N/A
Primer Il12a-Forward: AGACATCACACGGGACCAAAC	Microsynth	N/A
Primer IL12b-Forward: TGGTTTGCCATCGTTTTGCTG	Microsynth	N/A
Primer CD80-Forward: TCGTCTTTCACAAGTGTCTTCAG	Microsynth	N/A
Primer CD86-Forward: GAAGCCGAATCAGCCTAGC	Microsynth	N/A
Primer Gapdh-Forward: TGACCTCAACTACATGGTCTACA	Microsynth	N/A
GEFH1 guide RNA_1: GCACATGGTCATGCCGGAGA	Microsynth	N/A
GEFH1 guide RNA_2: GACAAGGTAGGAGTCAGCCT	Microsynth	N/A
Software and Algorithms		
Volocity	PerkinElmer	N/A
NIS-Elements imaging software	Nikon	N/A
ISMARA	https://ismara.unibas.ch	N/A
GraphPad Prism 7	GraphPad Software	N/A
FlowJo	https://www.flowjo.com/	N/A
Blc2fastq2 Conversion software	https://support.illumina.com/sequencing/sequencing_software/bcl2fastq-conversion-software.html	N/A
Cuffdiff version 1.05	https://software.broadinstitute.org/cancer/software/genepattern/modules/docs/Cuffdiff/7	N/A
Seqmonk	https://www.bioinformatics.babraham.ac.uk/projects/seqmonk/	N/A
STAR (2.5.2a)	Devred et al., 2010	N/A
edgeR Bioconductor Package in R	https://www.r-project.org	N/A
R package ComplexHeatmap	https://bioconductor.org/packages/release/bioc/html/ComplexHeatmap.html	N/A
HCOP: Orthology Predictions Search	http://www.genenames.org/cgi-bin/hcop at 8.9.17	N/A
GSEA Java application	http://software.broadinstitute.org/gsea/index.jsp	N/A
LM22 matrix	Newman et al., 2015	N/A
R package TCGAbiolinks	Colaprico et al., 2016	N/A
Cox regression analyses	Sauerbrei et al., 2007	N/A

## Highlights

- Microtubule destabilization in dendritic cells drives DC maturation and T cell activation
- GEF-H1 is released from microtubules, leading to its activation
- GEF-H1 release triggers the RhoA-JNK-c-Jun signaling axis and AP-1 transcriptional response
- GEF-H1 is critical for DC maturation, antigen cross-presentation, and anti-tumor immunity
