# Supplementary material for: GEF-H1 Signaling upon Microtubule Destabilization Is Required for Dendritic Cell Activation and Specific Anti-tumor Responses
Source: Cell Rep. Author manuscript; Available in PMC 2019 Nov 25. (PMC6876861; doi:10.1016/j.celrep.2019.08.057)
Supplement: 1 [file NIHMS1543785-supplement-1.pdf]

Cell Reports, Volume *28*

Supplemental Information

GEF-H1 Signaling upon Microtubule Destabilization Is Required for Dendritic Cell Activation and Specific Anti-tumor Responses

Abhishek S. Kashyap, Laura Fernandez-Rodriguez, Yun Zhao, Gianni Monaco, Marcel P. Trefny, Naohiro Yoshida, Kea Martin, Ashwani Sharma, Natacha Olieric, Pankaj Shah, Michal Stanczak, Nicole Kirchhammer, Sung-Moo Park, Sebastien Wieckowski, Heinz Laubli, Rachid Zagani, Benjamin Kasenda, Michel O. Steinmetz, Hans-Christian Reinecker, and Alfred Zippelius

GEFH1 signaling upon microtubule destabilization is required for dendritic cell activation and specific anti-tumor responses

Abhishek S Kashyap[1,2*], Laura Fernandez-Rodriguez[1#], Yun Zhao[2#], Gianni Monaco[1], Marcel P Trefny[1], Naohiro Yoshida[2], Kea Martin[1,6], Ashwani Sharma[3], Natacha Olieric[3], Pankaj Shah[2], Michal Stanczak[1], Nicole Kirchhammer[1], Sung-Moo Park[2], Sebastien Wieckowski[1,7], Heinz Laubli[1,4], Rachid Zagani[2], Benjamin Kasenda[4], Michel O Steinmetz[3,5], Hans-Christian Reinecker[2#*], Alfred Zippelius[1,4#*]

Supplementary Figures

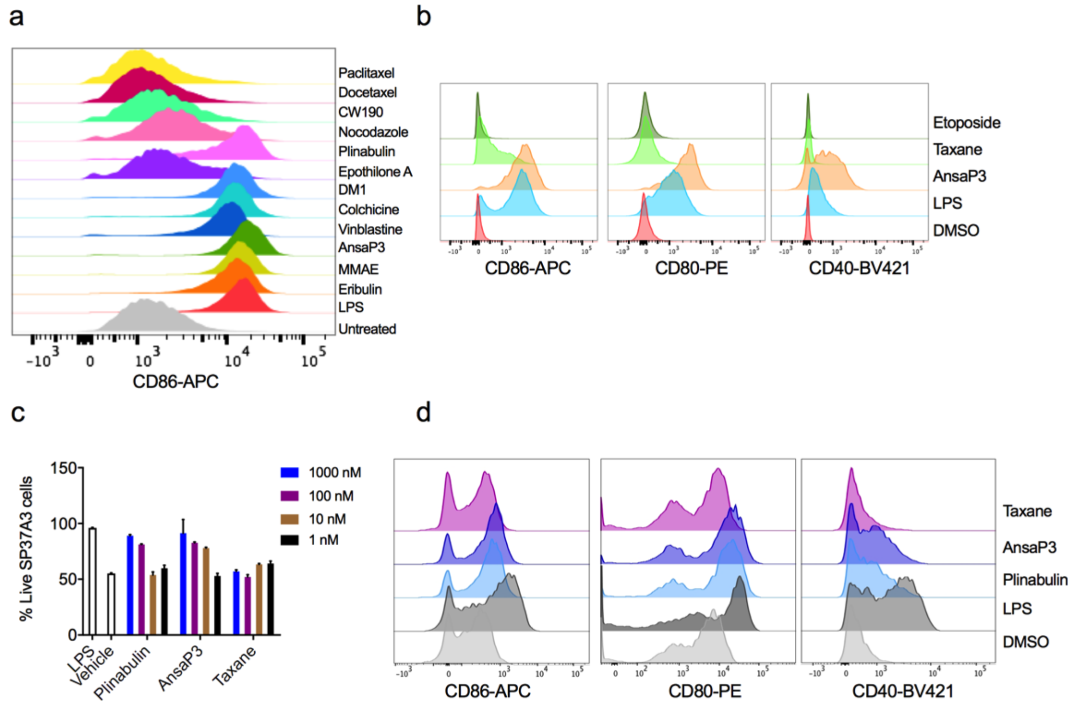

Supplementary Figure S1 (Related to Figure 1)

(a, b) Supplementary data for Figure 1a and 1c of main manuscript, respectively. Overlay of representative histograms for indicated proteins assessed by flow cytometry in SP37A3 cells treated for 20 hours with the corresponding drugs at 100 nM or LPS (500 ng/mL). **(c)** SP37A3 cells were treated with plinabulin, ansamitocin-P3 or taxane at the indicated doses for 20 hours after which cell viability was measured using the live/dead Zombie UV dye. LPS (500 ng/mL) and vehicle (0.1% DMSO) were the

controls. Data is expressed as percentage of live SP37A3 cells. Experiment was repeated three times with similar results. **(d)** Supplementary data for Figure 1d and 1e of main manuscript. Overlay of representative histograms for indicated proteins assessed by flow cytometry in splenic DCs treated with taxane (100 nM), MDAs ansamitocin-P3 (100 nM) or plinabulin (1000 nM) or LPS at 200 ng/mL. Error bars represent SD.

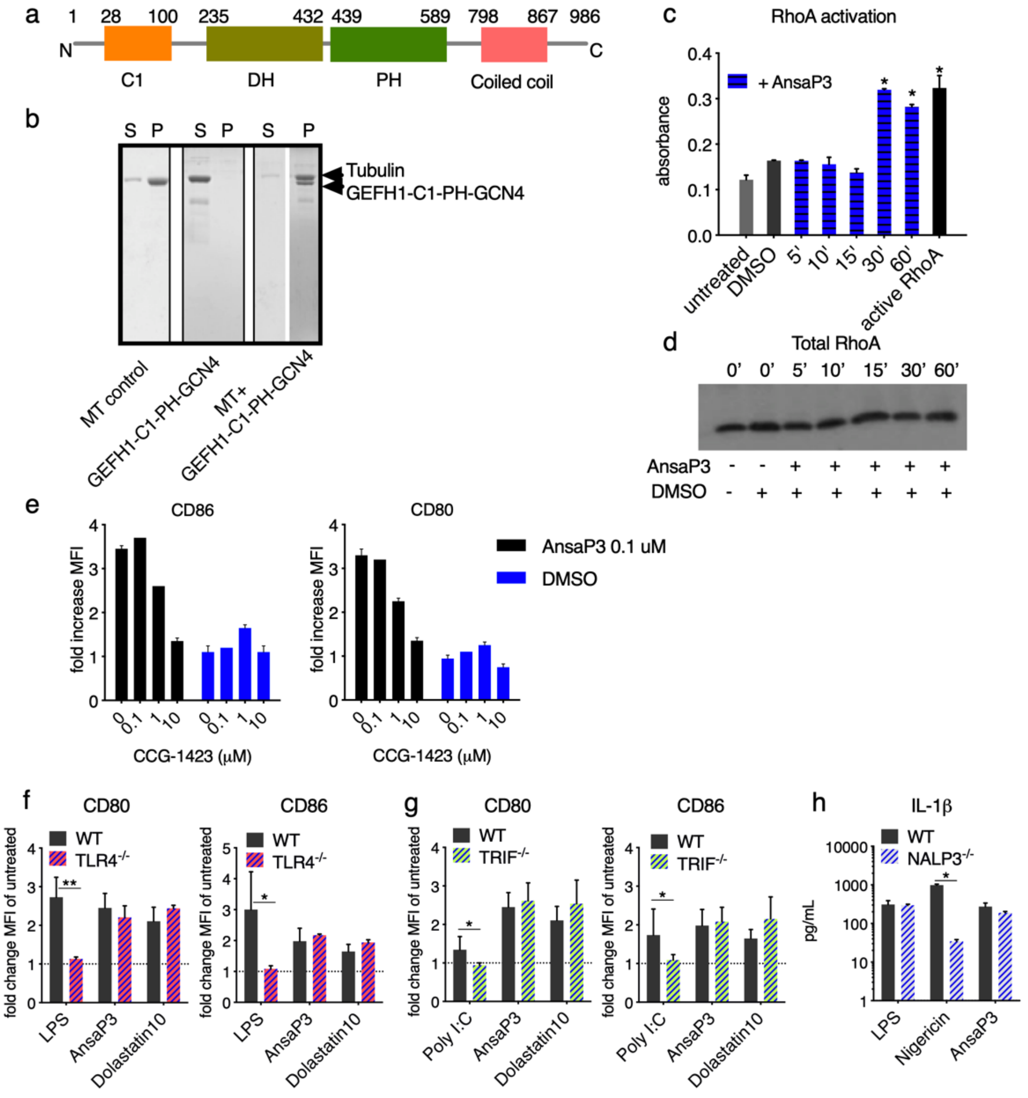

Supplementary Figure S2 (Related to Figure 2)

(a) Schematic representation of the domain organization of human GEFH1. Numbers above the schematic correspond to the amino-acids (DH: Dbl Homology; PH: Pleckstrin Homology). **(b)** Microtubule pelleting assays with microtubules alone (left panel), GEFH1-C1-PH-GCN4 alone (middle panel) and an equimolar mixture of microtubules and GEFH1-C1-PH-GCN4 (right panel). Shown are Coomasie stained 12% SDS-PAGE gels. S: supernatant; P: pellet; MT: microtubules. **(c)** SP37A3 DCs were incubated with ansamitocin-P3 (100 nM) for indicated time-points (in minutes) before collection of whole cell lysates. Rho-GTP in the lysate was measured using G-LISA. **(d)** Total cell

lysate from **c** was probed for RhoA using western blot to determine equal loading. Data is pooled from two independent experiments. **(e)** Serum-starved SP37A3 DCs were pretreated with the RhoA-inhibitor CCG-1423 at indicated concentrations (µM) for two hours before addition of ansamitocin P3 (0.1 µM) or DMSO control (0.1%) for another 18 h. MFI od CD80 and CD86 was assessed by flow cytometry; graphs show fold change of MFI compared with untreated cells, which were set as 1. **(f-h)** BMDCs from WT and the indicated KO mice were treated with MDAs ansamitocin-P3 or dolastatin 10 (100 nM) or controls for 24h. CD80 and CD86 expression was assessed by flow cytometry (fold change MFI compared to mock-treated cells) and IL-1β was measured by ELISA. LPS was the control for $TLR4^{-/-}$ **f**, Poly I:C for $TRIF^{-/-}$ **g**, and Nigercin for $NALP3^{-/-}$ **h**. Data is pooled from two independent experiments. Error bars represent SD.

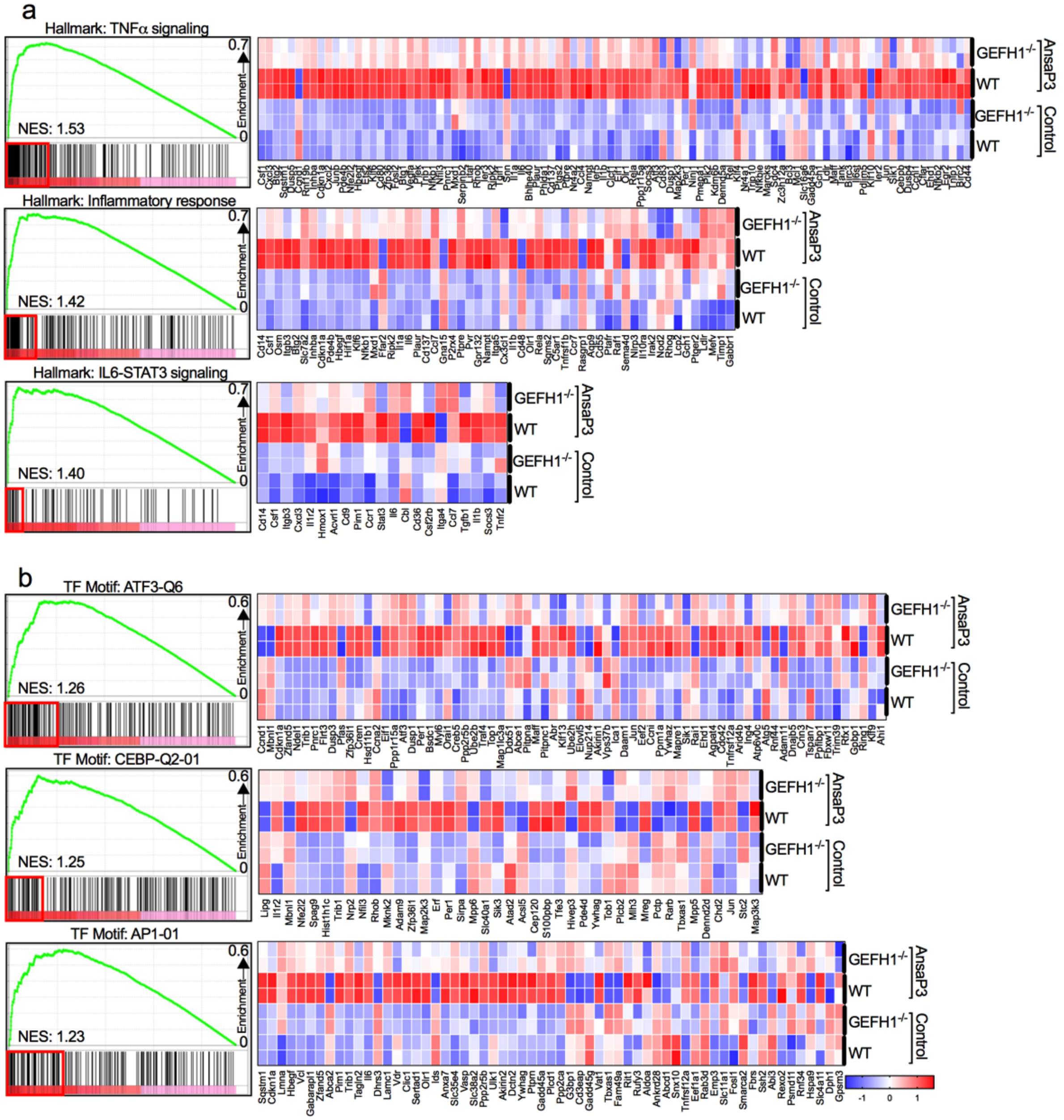

Supplementary Figure S3 (Related to Figure 3)

GSEA for differential up/down-regulation by ansamitocin-P3 in WT versus GEFH1$^{-/-}$ BMDCs was performed using ranked list of genes, generated according to the –log10 transformed corrected p-value for differential up/down-regulation by ansamitocin-P3 in WT versus GEFH1$^{-/-}$ BMDCs. Enrichment plots for three selected gene sets are shown for the Hallmark **a** and transcription factor motif **b** collection of MSigDB. NES is

indicated within the plot. Leading edge genes are shown as a heat map of scaled, centered logFPKM values across all samples.

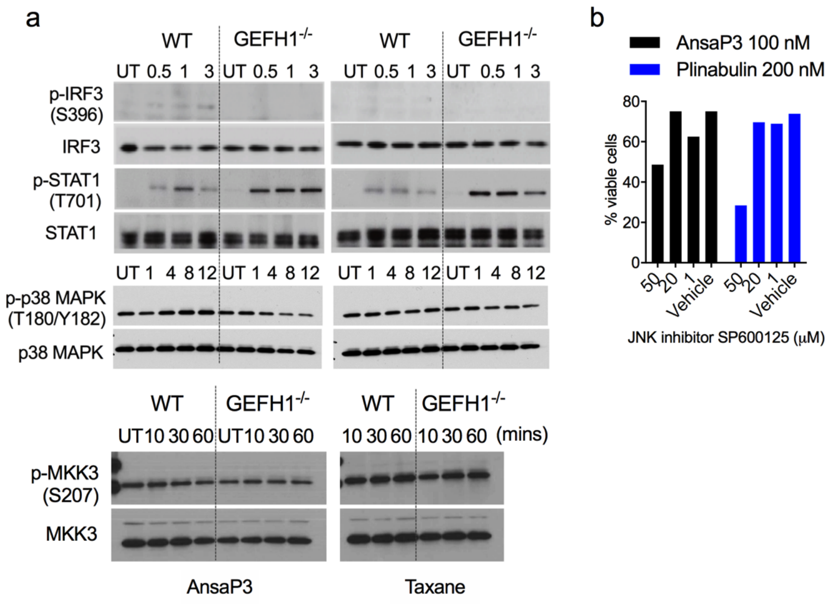

Supplementary Figure S4 (Related to Figure 4)

(a) Lysates from WT or GEFH1$^{-/-}$ BMDCs treated for specified time points (indicated in minutes) with ansamitocin-P3 (left) or taxane (right) at 100 nM were probed for phosphorylated IRF3, STAT1, p38 MAPK and MKK3. Blots were stripped and re-probed for the respective total proteins. (b) **(d)** DCs were pre-incubated with the indicated concentrations of the JNK inhibitor SP600125 or Vehicle (0.5% DMSO) for two hours after which they were exposed to MDAs ansamitocin-P3 (100 nM) or plinabulin (200 nM) for 20 hours. Cell viability was then assessed with Live/Dead Zombie UV dye.

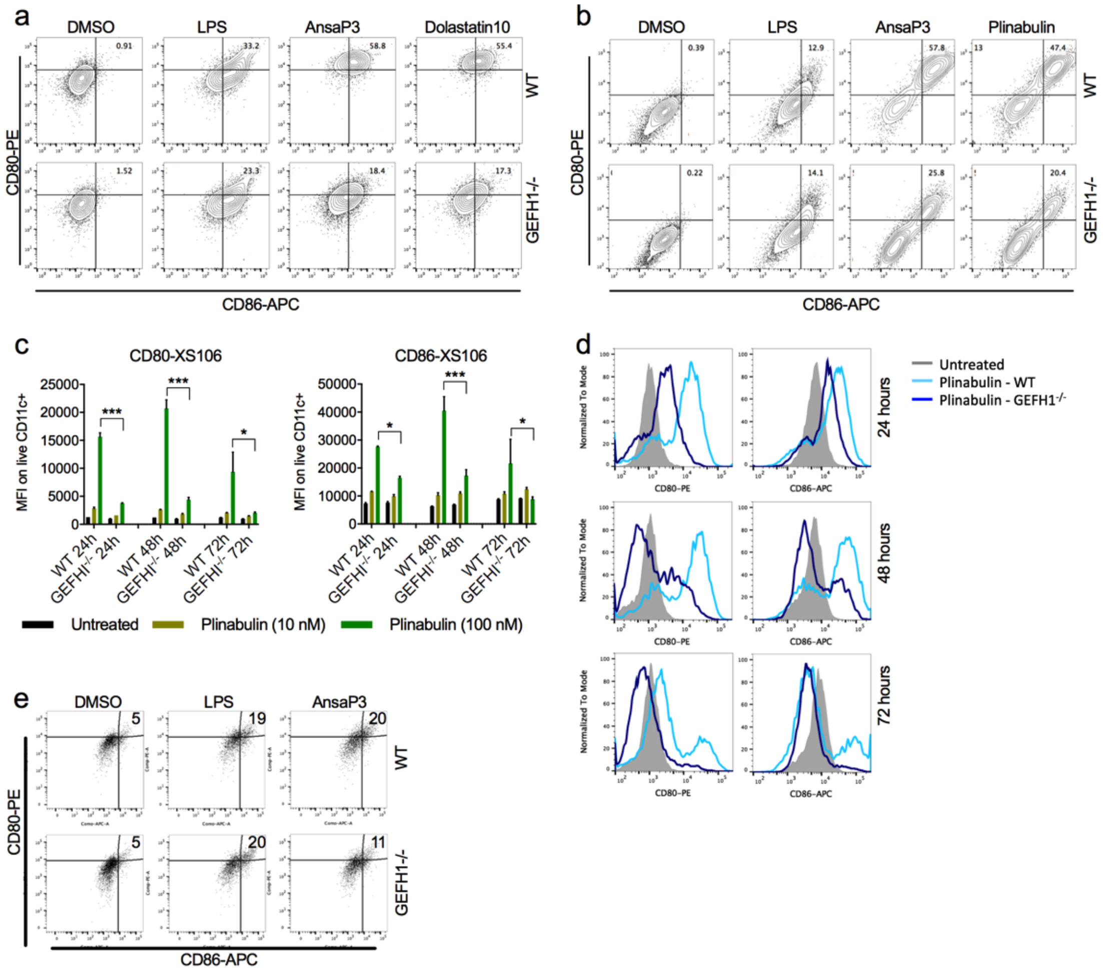

Supplementary Figure S5 (Related to Figure 5)

(a) Supplementary data for Figure 5c of main manuscript. BMDCs of WT or GEFH1$^{-/-}$ mice were stimulated with LPS (500 ng/mL), taxane, or MDAs ansamitocin-P3 and dolastatin 10 (all 100 nM) prior to assessment by flow cytometry (20 hours post stimulation). Percentages of gated populations of live CD11c+MHC-II+ BMDCs are indicated. **(b)** Supplementary data for Figure 5d of main manuscript. GEFH1$^{-/-}$ XS106 cells were stimulated with vehicle (0.1% DMSO), LPS (500 ng/mL), or MDAs ansamitocin-P3 and plinabulin (both 100 nM) prior to assessment by flow cytometry (20 hours post stimulation). Percentages of gated populations of live cells are indicated. **(c)** WT or GEFH1$^{-/-}$ XS106 cells were treated at indicated time points with plinabulin (10 nM and 100 nM). At endpoint, MFI for CD80 and CD86 was assessed by flow cytometry. **(d)**

Overlapping histograms from **c** are indicated for the 100 nM dose of plinabulin. **(e)** Supplementary data for Figure 5e of main manuscript. Ansamitocin-P3 (4 µg), LPS (8 µg) or vehicle alone (1.5% DMSO) was injected intradermal in the earflaps of WT and GEFH1$^{-/-}$ mice. CD80 and CD86 expression on intradermal CD11c^{+}MHC-II^{+} DCs was analyzed by flow cytometry. Error bars represent SD.

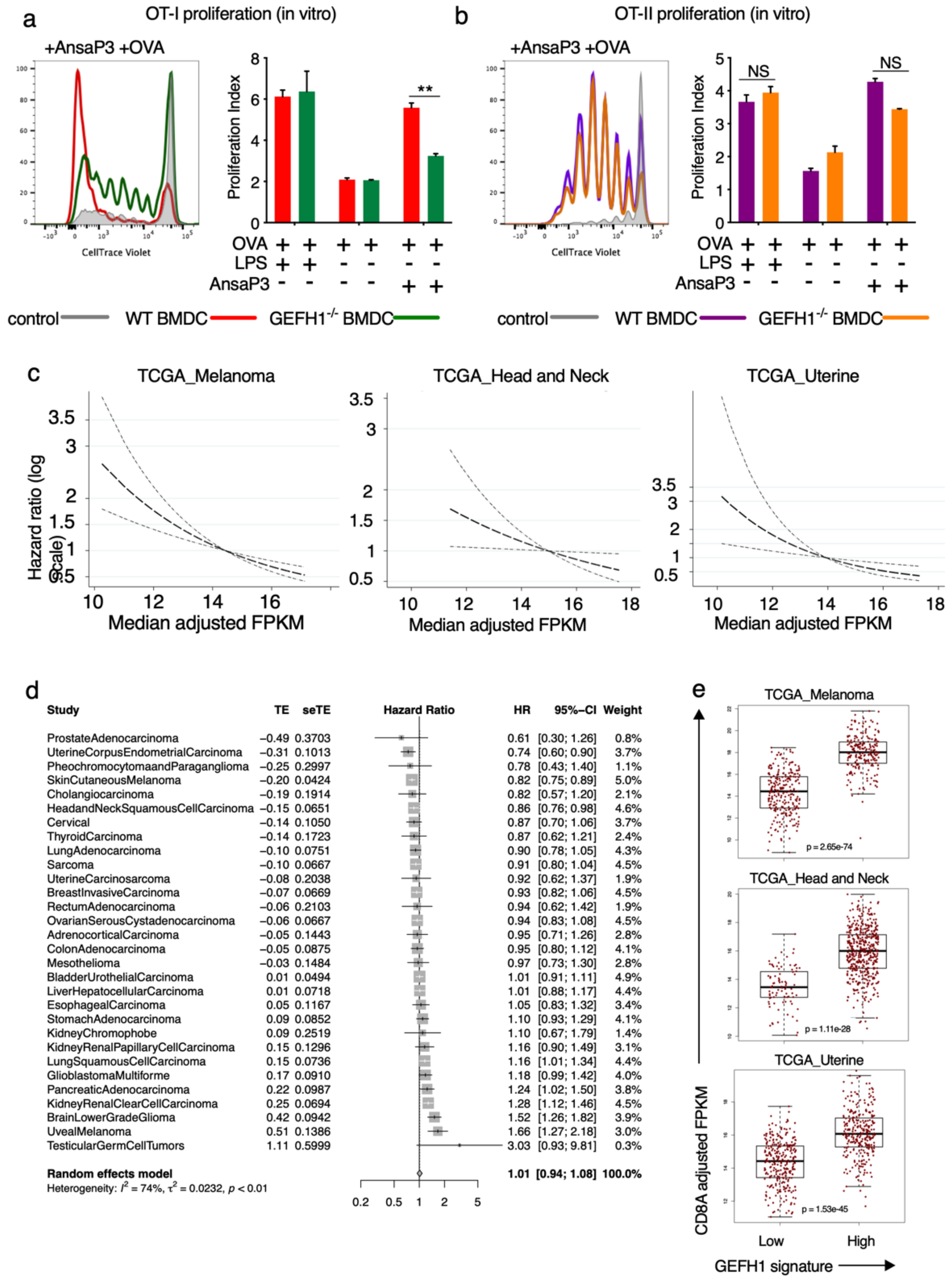

Supplementary Figure S6 (Related to Figure 6)

WT or GEFH1$^{-/-}$ BMDCs pre-treated with ansamitocin-P3 (100 nM), LPS (100 ng/mL) or vehicle alone (0.1% DMSO) and pulsed with OVA were co-cultured for 72 hours (without the drugs) with OT-I CD8+ **a** or OT-II CD4+ **b** cells pre-stained with CellTrace

Violet. Dye dilution was used to track up to 7 divisions from which the proliferation index was calculated. Bars indicate mean and SD pooled from two independent experiments. Representative histograms indicate overlap of dye dilution in ansamitocin-P3-treated DC:T cell co-culture using WT and GEFH1$^{-/-}$ BMDCs. Control (grey) histograms indicate OVA pulsed but untreated WT BMDCs co-cultured with OT-I/OT-II cells. Error bars represent SD. **(c)** Regression plots (effect with 95% confidence interval) depicting the association of increased expression levels of the GEFH1 Immune Signature with decreased risk of death. Graphs are depicted as hazard ratios versus median adjusted log2 FPKM of the GEFH1 Immune Signature. The upper confidence interval limit has a hazard ratio < 1 after logFPKM of 14. **(d)** Prognostic meta-analysis summarizing the prognostic effect of the GEFH1 immune signature in all solid tumors deposited in TCGA. A hazard ratio smaller than 1 implies a relative risk reduction of death. **(e)** CD8A expression in patient tumors stratified according to the median expression of GEFH1 Immune Signature (High: median log2 FPKM >=14; Low: median log2 FPKM <14).
